# Epithelial PBLD attenuates intestinal inflammatory response and improves intestinal barrier function by inhibiting NF-κB signaling

**DOI:** 10.1038/s41419-021-03843-0

**Published:** 2021-05-31

**Authors:** Shengbo Chen, Hongbin Liu, Zhijun Li, Jingyi Tang, Bing Huang, Fachao Zhi, Xinmei Zhao

**Affiliations:** Guangdong Provincial Key Laboratory of Gastroenterology, Institute of Gastroenterology of Guangdong Province, Department of Gastroenterology, Nanfang Hospital, Southern Medical University, 510515 Guangzhou, China

**Keywords:** Ulcerative colitis, Chronic inflammation

## Abstract

Intestinal barrier function defects and dysregulation of intestinal immune responses are two key contributory factors in the pathogenesis of ulcerative colitis (UC). Phenazine biosynthesis-like domain-containing protein (PBLD) was recently identified as a tumor suppressor in gastric cancer, hepatocellular carcinoma, and breast cancer; however, its role in UC remains unclear. Therefore, we analyzed colonic tissue samples from patients with UC and constructed specific intestinal epithelial PBLD-deficient (PBLD^IEC−/−^) mice to investigate the role of this protein in UC pathogenesis. We found that epithelial PBLD was decreased in patients with UC and was correlated with levels of tight junction (TJ) and inflammatory proteins. PBLD^IEC−/−^ mice were more susceptible to dextran sulfate sodium (DSS)- and 2,4,6-trinitrobenzene sulfonic acid-induced colitis compared with wild-type (WT) mice. In DSS-induced colitis, PBLD^IEC−/−^ mice had impaired intestinal barrier function and greater immune cell infiltration in colonic tissue than WT mice. Furthermore, TJ proteins were markedly reduced in PBLD^IEC−/−^ mice compared with WT mice with colitis. Nuclear factor (NF)-κB activation was markedly elevated and resulted in higher expression levels of downstream effectors (C–C motif chemokine ligand 20, interleukin [IL]-1β, IL-6, and tumor necrosis factor [TNF]-α) in colonic epithelial cells isolated from PBLD^IEC−/−^ mice than WT mice with colitis. PBLD overexpression in intestinal epithelial cells (IECs) consistently inhibited TNF-α/interferon-γ-induced intestinal barrier disruption and TNF-α-induced inflammatory responses via the suppression of NF-κB. In addition, IKK inhibition (IKK-16) rescued excessive inflammatory responses induced by TNF-α in PBLD knockdown FHC cells. Co-immunoprecipitation assays showed that PBLD may interact with IKKα and IKKβ, thus inhibiting NF-κB signaling, decreasing inflammatory mediator production, attenuating colonic inflammation, and improving intestinal barrier function. Modulating PBLD expression may provide a novel approach for treatment in patients with UC.

## Introduction

Ulcerative colitis (UC) is a nonspecific inflammatory bowel disease (IBD) of unknown cause, characterized by periods of relapse and remission and gastrointestinal symptoms, including bloody stool, diarrhea, and abdominal pain^1^. The global incidence of IBD has increased in the past few decades^2^. The highest incidence of UC was found on the Faroe Islands (57.9 per 100,000 persons)^3^. Sustained remission of UC only occurs in 20–30% of those with the disease when treated with biological agents targeting tumor necrosis factor (TNF)^4,5^. A greater understanding of the molecular mechanisms underlying the pathogenesis of UC could identify potential drug targets for the development of new therapeutic strategies.

Intestinal epithelial cells (IECs) regulate the intestinal barrier function and immune homeostasis^6^. Intestinal barrier dysfunction and dysregulated immune response are two key factors affecting the pathogenesis of UC^7,8^. Normally, the intestinal epithelial tight junction (TJ) serves as an physical barrier that limits the exposure of the mucosal immune system to pathogenic microorganisms^9^. Intestinal barrier dysfunction in UC includes decreased epithelial cell TJ resistance, increased intestinal permeability, and increased microbial exposure to the mucosal immune system, leading to uncontrolled inflammation^10^. Apart from their role as a barrier, IECs also secrete cytokines and chemokines (TNF-α, interleukin [IL]-1β, IL-8, and C–C motif chemokine ligand [CCL]-20) that mediate the interaction between the gut microbiome and mucosal immune system in response to pro-inflammatory cytokine stimuli or microbial invasion^6,11,12^. The secretion of these factors is primarily regulated by the nuclear factor (NF)-κB signaling pathway^13,14^. Taken together, previous research demonstrates the essential role of IECs in intestinal homeostasis; identifying the molecules that regulate IEC functions may therefore shed new light on the pathogenesis of UC.

Phenazine biosynthesis-like domain-containing protein (PBLD) is expressed in the liver, stomach, breast, kidney, and gut, and acts as a tumor suppressor in gastric cancer^15^, hepatocellular carcinoma^16,17^, and breast cancer^18^. The inhibitory role of PBLD in cancer is relatively clear; however, little is known about its function in inflammation, particularly in UC. In a previous study, we demonstrated that PBLD levels were significantly decreased in UC, and that the level of PBLD expression was negatively correlated with UC severity^19^, suggesting PBLD may play a role in the progression of this disease; however, the precise role of PBLD in the pathogenesis of UC remains poorly characterized. Microarray analysis has previously shown that PBLD acts as a tumor suppressor by inhibiting NF-κB and epithelial to mesenchymal transition signaling pathways^17^; NF-κB signaling is known to have a crucial role in cancer, and also acts as a key regulator in controlling inflammation. TNF-α, IL-1, and toll-like receptor ligand are known activators of NF-κB signaling; activation results in the phosphorylation of IκBα by the IκB kinase complex (IKK) and subsequent nuclear translocation of p65, which regulates the transcription of chemokines and cytokines^20^. NF-κB was found to be activated in IECs and in macrophages in the colonic mucosa of patients with UC^21^. Local administration of p65 antisense phosphorothioate oligonucleotides or a NF-kB decoy to block NF-κB signaling was shown to improve intestinal inflammation and restore tissue homeostasis in murine colitis^22–24^. Likewise, intraperitoneal injection of NF-κB essential modulator (NEMO)-binding domain peptide, which inhibits NF-κB activation, ameliorated murine colitis^25,26^. Preventing NF-κB activation may therefore be a promising therapeutic approach in patients with UC. Given the association of PBLD with UC severity, we hypothesized that PBLD may inhibit NF-κB activation and play a protective role in the pathogenesis of UC.

## Materials and methods

### Patient recruitment and collection of colonic tissue samples

Paired inflamed and non-inflamed colon tissue samples were collected from the same gut segment in nine patients with active UC and six patients with Crohn’s disease (CD) who underwent colonoscopy at the Department of Gastroenterology, Nanfang Hospital, China. The diagnosis of UC or CD was based on symptoms, endoscopy, histological examination, and lack of alternative diagnosis^7,8^. Demographic characteristics, including age, gender, disease severity, and treatment of these patients were recorded and shown in Supplementary Tables 1 and 2. The study was performed with the approval of the Institute Research Medical Ethics Committee of Nanfang Hospital (NFEC-2014-014) and conformed to the declaration of Helsinki. All patients gave written informed consent before the study.

### Cell culture and transfection

Three colonic cell lines, FHC, HT29, and Caco2, were purchased from the American Type Culture Collection (Manassas, VA). Cell lines were grown in Dulbecco’s modified Eagle’s medium (DMEM) containing 10% fetal bovine serum (FBS) at 37 °C and 5% CO_2_. All cell lines were authenticated using short tandem repeat profiling and were found to be without mycoplasma contamination.

A PBLD-expressing lentivirus was constructed by Genechem (Shanghai, China). FHC, HT29, and Caco2 cells were transfected with this lentivirus; cells that stably overexpressed PBLD were selected using puromycin.

FHC cell line was transfected with a small interfering RNA (siRNA; GenePharma, Shanghai, China) to knockdown PBLD expression. The specific siRNA sequences for PBLD were as follows: sense strand (5′–3′), GUAGAGGACUUGAUAAAGATT; antisense strand (5′–3′), UCUUUAUCAAGUCCUCUACTT.

### Immunohistochemical and immunofluorescence assays

The mouse colon tissue was fixed in 4% paraformaldehyde for 24 h and then embedded in paraffin; then a 4 µm thickness colon tissue sections were cut for immunohistochemistry and immunofluorescence. For immunohistochemical staining, the colonic sections were performed deparaffinization, hydration, antigen retrieval, quenching of endogenous peroxidase, and blocking procedures. All slices were then incubated with the primary antibodies against PBLD, MUC2, zonula occludens (ZO)-1, Occudin, myeloperoxidase (MPO), and F4/80 (Supplementary Table 3) at 4 °C overnight followed by incubation with biotinylated secondary antibodies for 30 min and visualization using a 3,3′-diaminobenzidine kit (ZSGB-BIO, Beijing, China). For immunofluorescence staining, the colonic sections were proceeded as the above steps; secondary (indirect) immunofluorescent staining was conducted using goat anti-rabbit IgG/Cy3 (Bioss, Beijing, China) or Alexa Fluor 488-labeled goat anti-rabbit IgG (Beyotime, Shanghai, China) antibodies.

For cell immunofluorescence, HT29 and FHC cells were fixed with 4% paraformaldehyde for 20 min; Caco2 monolayers were fixed with methanol for 10 min at −20 °C. Permeabilization and blocking was carried out with 0.3% Triton X-100 and 5% bovine serum albumin in phosphate-buffered saline (PBS) for 1 h at room temperature. Cells were then incubated with primary antibodies against P65, ZO-1, and Occludin at 4 °C overnight. After washing with PBS, cells were incubated with goat anti-rabbit IgG/Cy3 secondary antibodies in the dark for 1 h, followed by 4′,6-diamidino-2-phenylindole staining, and were analyzed using confocal microscopy.

### Co-immunoprecipitation and western blotting assays

For co-immunoprecipitation, protein lysates of FHC cells were prepared according to previously established methods^27^. Protein lysates (1000 μg) were incubated with 2 μg of the indicated antibody or the control IgG antibody and 20 μL protein A/G magnetic beads (Bimake, Houston, USA) overnight at 4 °C. After washing three times with lysis buffer, protein–antibody immune complexes were analyzed using western blots with the indicated primary antibodies (Supplementary Table 3). Western blot procedures were performed according to previously published methods^27^.

### Quantitative reverse-transcription polymerase chain reaction (qRT-PCR)

qRT-PCR was performed according to previously published methods^27^. All mRNA levels were normalized against glyceraldehyde 3-phosphate dehydrogenase (GAPDH) mRNA. The primers that were used in qRT-PCR are listed in Supplementary Table 4.

### Transepithelial electrical resistance (TEER) measurements and in vitro cellular permeability study

TEER was measured in Caco2 monolayers as described in previous methods^28^. In vitro cellular permeability studies in Caco2 monolayer were also performed as described in previous work^29^.

### Generation of PBLD^IEC−/−^ mice

Both *Pbld1* and *Pbld*2 genes encode PBLD proteins in mice. A murine strain with *Pbld1* and *Pbld2* flanked by *LoxP* (PBLD^FL/FL^) was constructed using the CRISPR/Cas9 gene editing system. Briefly, Cas9 mRNA, sgRNA, and donor vectors were co-injected into zygotes. sgRNA induced Cas9 endonuclease cleavage upstream of *Pbld2* exon 3 and downstream of the *Pbld1* 3′ untranslated region (UTR), creating two double-strand breaks. The repair of these breaks resulted in in *LoxP* sites inserted in the upstream region of *Pbld2* exon 3 and downstream of the *Pbld1* 3′UTR, respectively, by homologous recombination (Supplementary Fig. 1a and Supplementary Table 5). In order to generate the PBLD^IEC−/−^ mice, female PBLD^FL/FL^ mice were crossed with male Villin-Cre transgenic mice, expressing Cre recombinase under the control of the villin gene regulatory region. The resultant PBLD^FL/FL^ mice expressing the Cre gene were PBLD^IEC−/−^ mice, and PBLD^FL/FL^ cohoused littermates without the Cre gene were used as control (marked as wild type (WT) in all experiments for convenience, but were not exactly WT). All mice were backcrossed with the C57BL/6 inbred laboratory mouse strain for eight generations. All mouse experiments were approved by the Experimental Animal Ethics Committee of Southern Medical University (2016081) and in compliance with institutional regulations.

### Dextran sulfate sodium (DSS)-induced colitis

DSS-induced colitis is a widely used method for experimentally inducing colitis that closely mimics UC in humans based on the key immunological and histopathological characteristics; therefore, we used this disease model in our study^30^. To induce acute colitis in mice, 8-week-old PBLD^IEC−/−^ and WT littermates were fed with 2.5% DSS dissolved in drinking water for 5 days, followed by 3 days of regular drinking water. Disease activity index (DAI) was assessed daily; the details of this index are shown in Supplementary Table 6. To induce chronic colitis, PBLD^IEC−/−^ and WT littermates were fed with 2% DSS in drinking water for 7 days followed by 14 days regular drinking water; this cycle was repeated twice more for a total of three cycles.

### 2,4,6-Trinitrobenzene sulfonic acid (TNBS)-induced colitis

The TNBS-induced colitis model shares some immunological and histopathological features with CD; thus, we used this model to evaluate the role of PBLD in the pathogenesis of CD, to complement our mouse model of UC. Colitis was induced in 8-week-old PBLD^IEC−/−^ and WT littermates using TNBS, as previously described^30^. Briefly, mice were sensitized with putting 1% TNBS solution on their back. After 7 days, each mouse was given 2.5% TNBS solution by enema. The body weight of each mouse was measured each day for 2 days, after which mice were sacrificed for further examination.

### Enzyme-linked immunosorbent assay (ELISA)

Mice colon tissue samples were homogenized in lysing buffer containing a protease inhibitor, and protein concentrations were determined using a bicinchoninic acid assay. The levels of TNF-α, IL-6, and interferon (IFN)-γ in mice colon tissues were detected using ELISA kits (Invitrogen, Carlsbad, CA), according to the manufacturer’s guidelines.

### Isolation of primary colonic epithelial cells and colonic lamina propria (CLP) cells from mice

Freshly harvested colonic tissue from mice was washed with PBS, cut into 1 × 1 cm segments, and incubated with DMEM containing 3% FBS, 5 mM ethylenediaminetetraacetic acid and 1 mM dithiothreitol for 30 min at 37 °C with shaking at 200 r.p.m. The supernatant was filtered through a 70 μm cell strainer, and colonic epithelial cells were collected by centrifugation at 1000 × *g* for 10 min at 4 °C. The colon fragments were then incubated with DMEM supplemented with 3% FBS and 500 μg/mL collagenase type IV (Sigma) and 0.5 U/mL Dispase II (Yeasen) at 37 °C for 30 min with shaking at 80 r.p.m. The supernatant was again filtered through a 70 μm cell strainer, followed by centrifugation at 450 × *g* for 5 min; the resulting sediment was purified using 40/80% discontinuous Percoll gradient centrifugation. After centrifugation at 2500 r.p.m. for 20 min, CLP cells were obtained at the interface of the Percoll gradient.

### Flow cytometry

To evaluate immune cell infiltration in the CLP and mesenteric lymph nodes (MLN), WT and PBLD^IEC−/−^ mice were given 2.5% DSS drinking water for 5 days, and sacrificed to isolate CLP and MLN cells. Isolated CLP and MLN cells were stained with the indicated antibodies (Supplementary Table 3). Flow cytometry analysis was performed using a flow cytometer (Aria II, BD bioscience), and the gating strategy outlined in Supplementary Figs. 2 and 3. Data were analyzed by FlowJo-v10.6.2 software.

### Intestinal permeability assay

An assay to evaluate intestinal permeability in mice was performed using a previously established protocol^28^.

### Histological analysis

Tissue samples from mice with DSS-induced colitis were evaluated and scored in a blinded manner on the basis of previously defined criteria^27^. The histologic scores in TNBS-induced colitis mice were also assessed as previously described^30^.

### Statistical analysis

All data are presented as mean ± standard deviation (SD). Graph Pad Prism 8 software were used for statistical analysis. Student’s *t* test, one-way analysis of variance (ANOVA) or two-way ANOVA, as appropriate, were used to determine significant differences between groups. All statistical tests were two-tailed and a *p* value of <0.05 was considered significant.

## Results

### PBLD expression is decreased in patients with UC

**Fig. 1:**
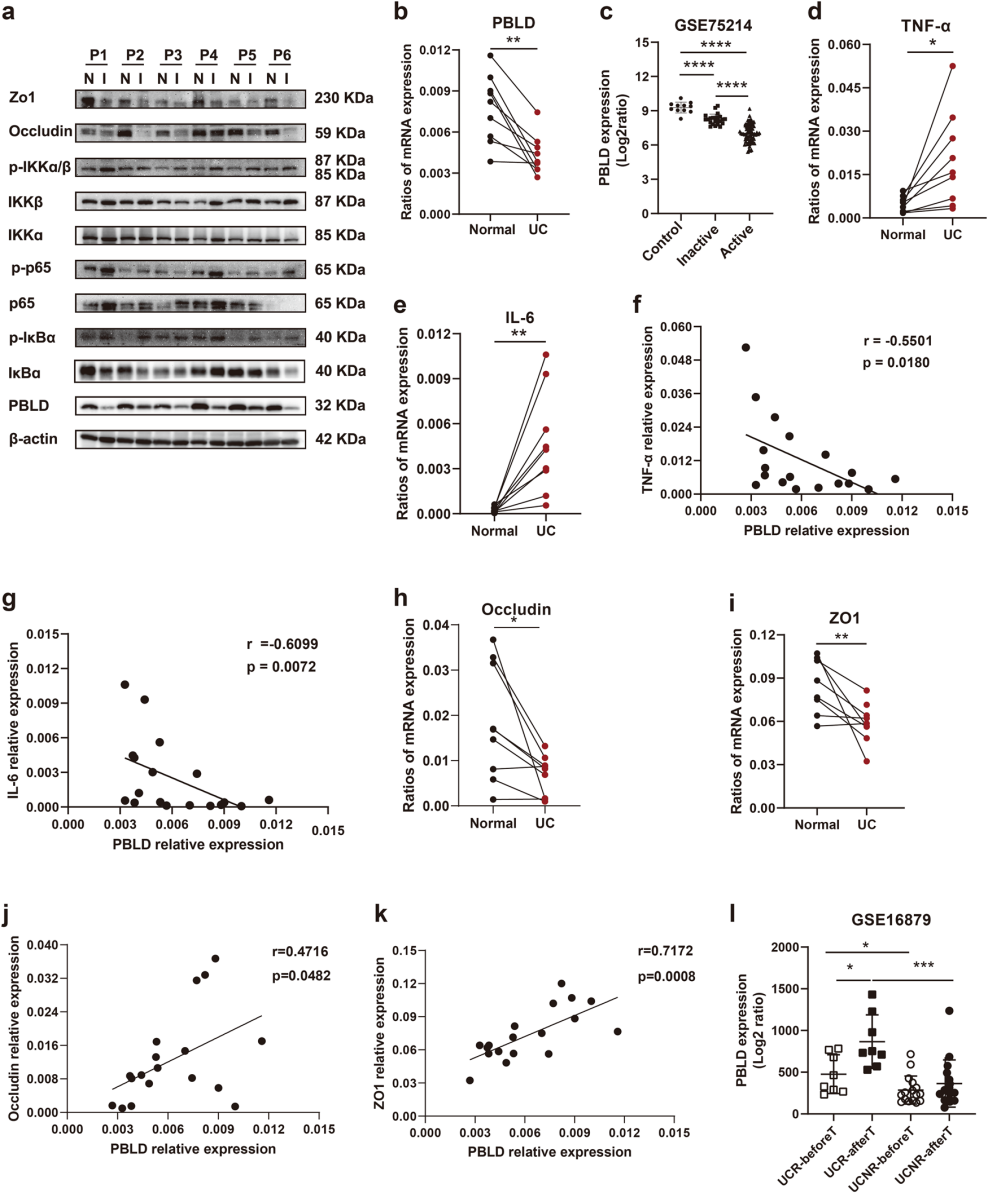
PBLD is decreased in people with UC. **a** Expression of PBLD, tight junction (TJ) proteins, and the nuclear factor (NF)-κB signaling pathway was evaluated by immunoblotting in paired colon tissues from patients with UC (*n* = 6). β-actin served as an internal control. N normal colon tissue, I inflamed colon tissue. **b** Expression of PBLD mRNA was analyzed by quantitative reverse-transcriptase (qRT)-PCR in paired inflamed and non-inflamed colon tissue samples from UC patients (*n* = 9). Glyceraldehyde 3-phosphate dehydrogenase (GAPDH) was used as an internal control. **p < 0.01. **c** Expression of PBLD mRNA was assessed using a published dataset (GSE75214; *N* = 108; normal controls, *n* = 11; inactive UC, *n* = 23; active UC, *n* = 74) ****p < 0.0001. **d**, **e** Levels of TNF-α and IL-6 mRNA were analyzed using qRT-PCR in paired inflamed and non-inflamed colon tissue samples from patiens with UC (*n* = 9). **f** Spearman’s correlation analysis between PBLD and TNF-α mRNA levels (*r* = −0.5501, *p* = 0.0180). **g** Spearman’s correlation analysis between PBLD and IL-6 mRNA levels (*r* = −0.6099, *p* = 0.0072). **h**, **i** Occludin and ZO-1 mRNA levels were analyzed using qRT-PCR in paired inflamed and non-inflamed colon tissue samples from patients with UC (*n* = 9). **j** Spearman’s correlation between PBLD and occludin mRNA levels (*r* = 0.4716, *p* = 0.0482). **k** Spearman’s correlation between PBLD and ZO-1 mRNA levels (*r* = 0.7172, *p* = 0.0008). **l** PBLD mRNA levels were analyzed using a published dataset from patients with UC who received infliximab treatment. (GSE16879; *N* = 24; those who responded to treatment, *n* = 8; nonresponders, *n* = 16). UCR-beforeT responder before infliximab treatment, UCR-afterT responder after infliximab treatment, UCNR-beforeT nonresponder before infliximab treatment, UCNR-afterT nonresponder after infliximab treatment.

We compared PBLD expression of paired inflamed and non-inflamed colonic tissue samples taken from people with UC, and found that PBLD mRNA and protein levels were significantly decreased in inflamed colonic tissue samples compared with non-inflamed samples (Fig. 1a, b). This result is consistent with data from a public dataset of UC samples (GSE75214), which shows significantly reduced PBLD mRNA levels in patients with UC, especially in those with active disease (Fig. 1c).

In addition, we observed that expression of TNF-α and IL-6 mRNA was significantly increased in inflamed colonic tissue samples from people with UC compared with samples with no inflammation (Fig. 1d, e). Spearman correlation analysis revealed that PBLD mRNA levels were negatively correlated with TNF-α and IL-6 mRNA levels in colonic tissue samples from people with UC (Fig. 1f, g), indicating a role for PBLD in inflammation. The expression of p-p65, p-IκBα, and p-IKKα/β was also increased in inflamed versus non-inflamed colonic tissue samples, indicating activation of the NF-κB pathway (Fig. 1a and Supplementary Fig. 4).

The TJ plays important role in the pathogenesis of UC; we therefore evaluated the expression of TJ proteins, and found that ZO-1 and occludin levels were reduced in inflamed tissue samples compared with non-inflamed samples (Fig. 1a, h, i), which was consistent with previously published work^31^. Spearman correlation analysis revealed that both ZO-1 and occludin mRNA levels were positively correlated with those of PBLD in tissue samples from patients with UC (Fig. 1j, k).

To further assess the relationship between PBLD and TNF-α, we analyzed their expression levels in samples taken after infliximab (IFX) anti-TNF-α induction therapy, using publicly available data (from the UC sample dataset GSE16879). Compared with those who did not respond to IFX therapy, responders had higher PBLD levels in colon mucosal tissue before treatment, and IFX treatment further increased PBLD expression (Fig. 1l), indicating that PBLD may act as a potential biomarker for response to anti-TNF-α treatment in UC patients.

We also evaluated the expression of PBLD and occludin in the paired inflamed and non-inflamed colon tissue of people with CD. Similar to the findings in those with UC, PBLD and occludin levels were decreased in inflamed colon tissues compared with non-inflamed colon tissue (Supplementary Fig. 5). These results indicate that decreased expression of PBLD may be involved in the pathogenesis of IBD, rather than just UC.

### Colonic function of epithelial PBLD knockout mice is normal under baseline conditions

We explored the function of epithelial PBLD levels in UC using a PBLD^IEC−/−^ mouse strain. Western blot analysis confirmed that PBLD was ablated in the small intestine, colonic tissue, and epithelial extracts from the colon, but not in liver tissue (Supplementary Fig. 1b). To determine whether the ablation of intestinal epithelial PBLD resulted in spontaneous colitis, we measured colon length, histological structure, distribution pattern of goblet cells, and proliferative activity in the colonic tissue samples. PBLD^IEC−/−^ mice had similar colon lengths to WT mice (Supplementary Fig. 1c). In addition, hematoxylin–eosin (HE) staining did not reveal any obvious differences in histological structure; similarly, periodic acid–Schiff staining did not indicate any differences in goblet cell distribution between PBLD^IEC−/−^ and WT mice. Ki67 staining showed that proliferating cells were similar between PBLD^IEC−/−^ and WT mice (Supplementary Fig. 1d). Collectively, these results demonstrate that ablating PBLD expression in the intestinal epithelium of mice does not affect normal colonic functions under baseline conditions, in the absence of colitis.

### Deletion of PBLD in IECs aggravates DSS- and TNBS-induced colitis

In our study, DSS-induced colitis was used as a mouse model of UC to explore the role of PBLD in the pathogenesis of this disease. Consistent with the observation in colonic tissue from people with UC, the expression of PBLD was decreased in DSS-induced acute colitis in mice compared with control mice. (Supplementary Fig. 6a). This finding was supported by immunochemical staining that showed PBLD was primarily expressed in epithelial cells (Supplementary Fig. 6b). The abundance of PBLD was also significantly higher in epithelial cells than in CLP cells from the mouse colon tissue samples (Supplementary Fig. 6c).

**Fig. 2:**
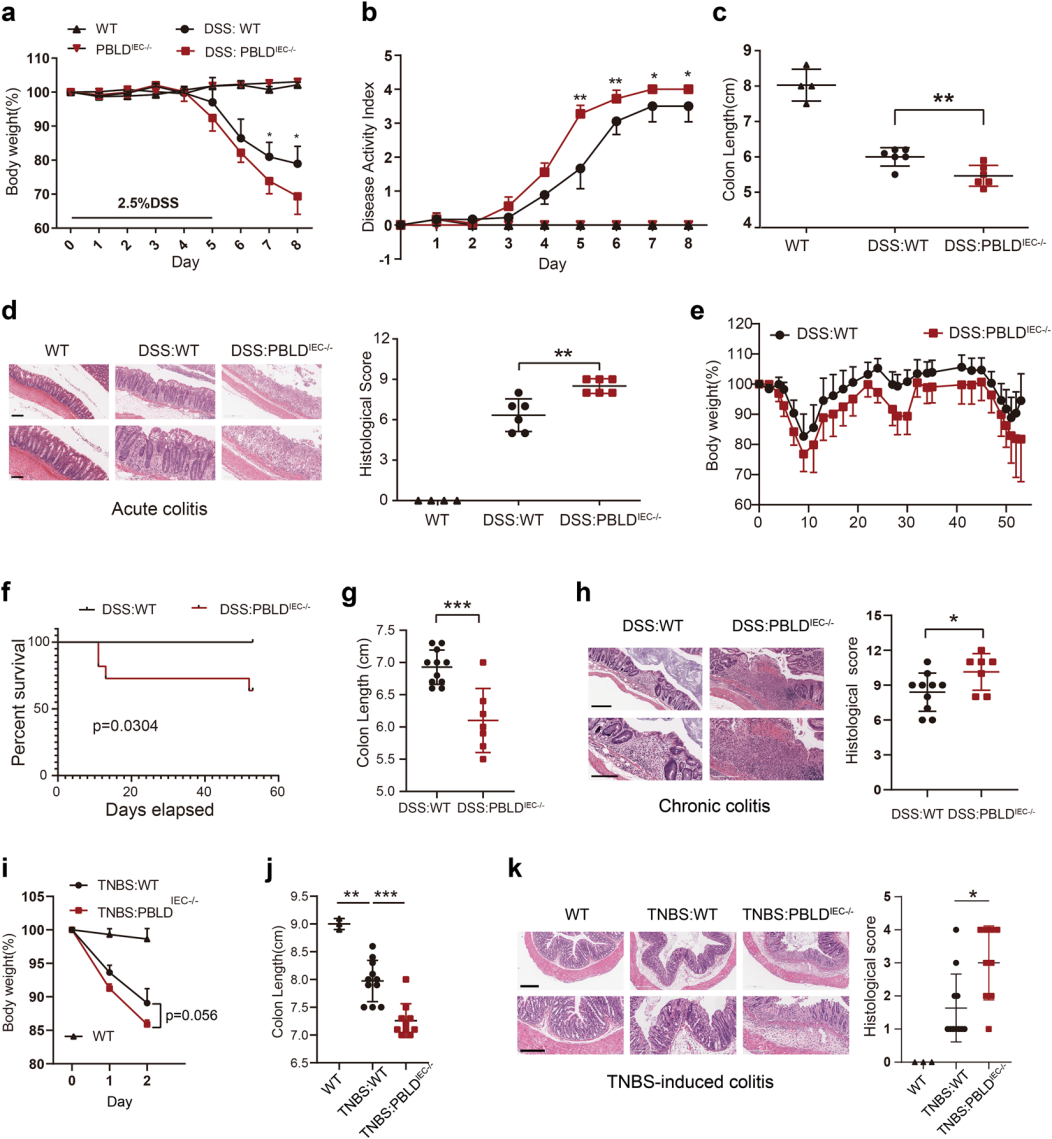
Deletion of PBLD in IECs aggravates DSS- and TNBS-induced colitis in mice. **a** Body weight curve and **b** disease activity index of wild-type (WT) and PBLD^IEC−/−^ mice treated with DSS for 5 days, followed by 3 days regular drinking water (WT, *n* = 4; PBLD ^IEC−/−^, *n* = 4; DSS:WT, n = 6; DSS:PBLD^IEC−/−^, *n* = 6). **c** Colon length of mice sacrificed at day 8. **d** Representative HE-stained colon sections in mice (left) and their corresponding histological score (right) (*n* = 4–6). Scale bars: 200 μm in upper,100 μm in lower. **e** Body weight curve and **f** survival curve in the DSS-induced chronic colitis model in WT and PBLD^IEC−/−^ mice (*n* = 11 in each group). **g** Colon length of mice sacrificed at the end of chronic colitis model (*n* = 7–11). **h** Representative HE-stained colon sections in mice with chronic colitis (left) and their corresponding histological scores (right; *n* = 7–10). Scale bars: 300 μm in upper, 200 μm in lower. Data are mean ± standard deviation (SD). ***p < 0.001. **i** Body weight changes in WT mice and PBLD ^IEC−/−^ mice after TNBS administration. **j** Colon length in mice after 2 days of TNBS treatment (*n* = 3–11). **k** HE-stained colon sections in mice after 2 days of TNBS treatment (left) and their corresponding histological scores (right). Scale bars: 300 μm in upper, 200 μm in lower.

In the DSS-induced acute colitis model, we found that PBLD^IEC−/−^ mice lost more body weight than WT mice (Fig. 2a), and the DAI score in the PBLD^IEC−/−^ mice was much higher (Fig. 2b). Furthermore, our dissections revealed that colon length was significantly reduced in these mice compared with their WT counterparts (Fig. 2c). Histopathological examination revealed that PBLD^IEC−/−^ mice with DSS-induced colitis showed extensive epithelial denudation and more ulcers in the colonic epithelium compared with the WT mice (Fig. 2d).

In accordance with our results for the acute colitis model, epithelial PBLD deficiency also exacerbated disease severity in the chronic colitis model in mice. During DSS-induced chronic colitis, PBLD-deficient mice lost more body weight (Fig. 2e) and were more prone to early death compared with their WT counterparts, all of which survived through three cycles of DSS treatment (Fig. 2f). Moreover, as in the acute colitis model, colon length in PBLD-deficient mice was significantly shorter and the histological score was significantly higher than in the WT mice (Fig. 2g, h).

TNBS-induced colitis in mice was used as a model for CD in our study. We found that PBLD^IEC−/−^ mice were more susceptible to TNBS-induced colitis compared with their WT counterparts. PBLD^IEC−/−^ mice lost more body weight than WT mice, though this result was not statistically significant (Fig. 2i). However, colon length in PBLD^IEC−/−^ mice was significantly shorter than that of WT mice in TNBS-induced colitis (Fig. 2j); the PBLD^IEC−/−^ mice had a higher histological score in colonic tissue than the WT mice (Fig. 2k). Taken together, these results indicate that epithelial PBLD may have a protective role in both DSS- and TNBS-induced colitis.

### Loss of PBLD expression in IECs impairs intestinal barrier function in DSS-induced colitis

**Fig. 3:**
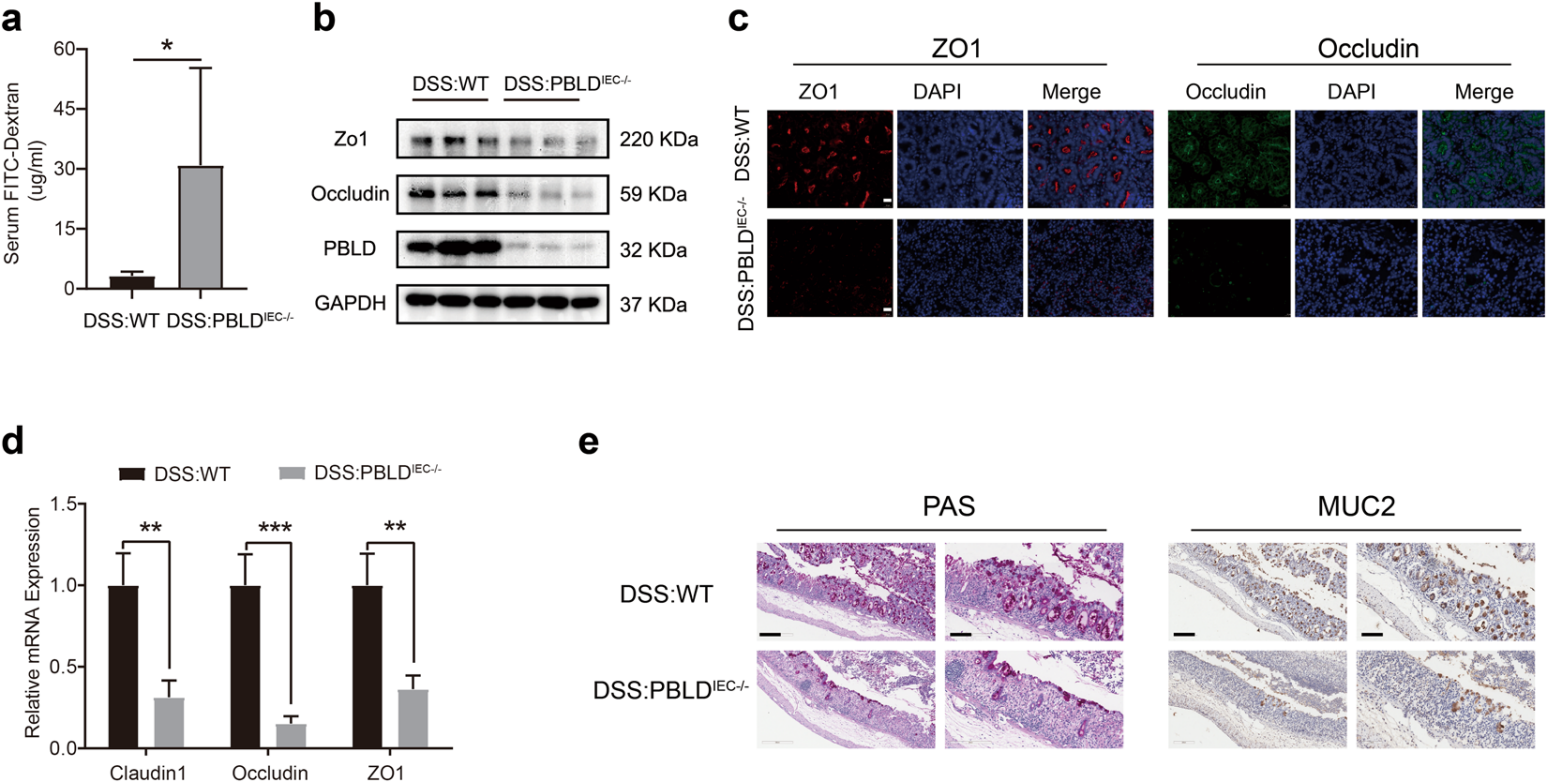
Epithelial PBLD deficiency impairs intestinal barrier function in mice with DSS-induced colitis. **a** Intestinal permeability was evaluated by measuring the concentration of fluorescein isothiocyanate (FITC)-dextran in the blood serum of wild-type (WT) and PBLD^IEC−/−^ mice after treatment with DSS for 5 days followed by 3 days regular drinking water (*n* = 6). Data are mean ± standard deviation (SD). *p < 0.05. **b** Western blot analysis of proteins in whole-colon tissue samples from mice with DSS-induced colitis. **c** Immunofluorescent staining of ZO-1 and occludin in colonic sections from WT and PBLD^IEC−/−^ mice with colitis. Scale bars: 50 μm. **d** qRT-PCR analysis of claudin-1, occludin, and ZO-1 mRNA levels in colonic tissue samples from WT and PBLD^IEC−/−^ mice with colitis (*n* = 4). Data are mean ± SD. ***p < 0.001. **e** Periodic acid–Schiff (PAS) and mucin 2 (MUC2) staining of colonic sections from WT and PBLD^IEC−/−^ mice with colitis. Images are representative from four mice in each group. Scale bars: 200 μm in left, 100 μm in right.

Intestinal barrier dysregulation is a key factor contributing to the pathogenesis of UC^10^. We therefore compared the intestinal permeability of PBLD^IEC−/−^ mice with WT mice in our DSS-induced colitis model by measuring FITC-dextran concentrations in serum following oral administration. We found that the serum concentration of FITC-dextran in PBLD^IEC−/−^ mice was significantly higher than that in WT mice (Fig. 3a), suggesting that epithelial PBLD deficiency results in increased intestinal permeability in DSS-induced colitis. Intestinal permeability is mainly regulated by TJ proteins, consisting of ZO-1, occludin, and claudin-1 (ref. ^32^). We found that ZO-1 and occludin expression was markedly decreased in PBLD^IEC−/−^ mice compared with WT mice in DSS-induced colitis (Fig. 3b and Supplementary Fig. 7). Immunofluorescent staining and qRT-PCR results confirmed this finding (Fig. 3c, d). Goblet cells, which secrete mucus to protect the epithelium and thus play a crucial part in maintaining intestinal barrier function^33^, were also significantly decreased in the colonic epithelium of PBLD^IEC−/−^ mice compared with WT mice subjected to DSS (Fig. 3e). Collectively, these results indicate that PBLD deficiency in IECs impairs intestinal barrier function in DSS-induced colitis.

### PBLD improves epithelial barrier function in a Caco2 cell monolayer by modulating the expression of TJ proteins

**Fig. 4:**
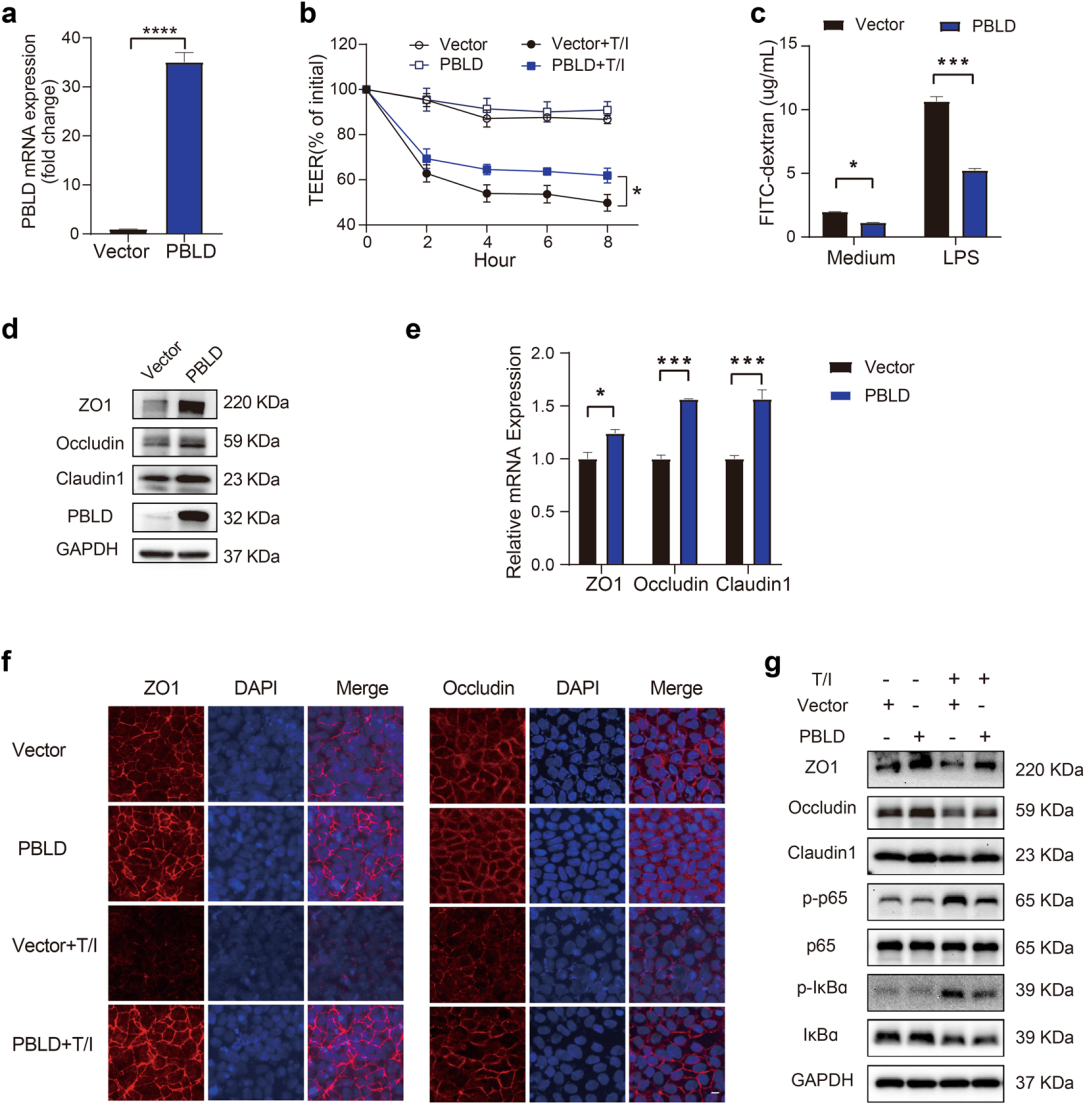
PBLD protects epithelial barrier function in Caco2 cell monolayers by improving TJ protein expression. **a** Caco2 cells were transfected with PBLD-expressing lentivirus or control lentivirus vector, then the expression of PBLD was assessed by qRT-PCR. **b** Transepithelial electrical resistance (TEER) curves from Caco2 monolayers transfected with PBLD-expressing lentivirus or control lentivirus vector with or without incubation with 10 ng/mL TNF-α/IFN-γ. **c** Caco2 monolayers transfected with PBLD-expressing lentivirus or control lentivirus vector was incubated with or without 50 ng/mL LPS for 2 h, then FITC-dextran flux was measured after 2 h incubation in upper chamber. **d** Immunoblotting analysis and (**e**) qRT-PCR analysis of proteins and genes in Caco2 monolayers transfected with PBLD-expressing lentivirus or control lentivirus vector. **f** Immunofluorescent staining of ZO-1 and occludin in Caco2 monolayers transfected with PBLD-expressing lentivirus or control lentivirus vector with or without 10 ng/mL TNF-α/IFN-γ incubation for 24 h. Scale bars: 10 μm. **g** Western blot analysis of indicated proteins in Caco2 monolayers, as described in (**f**).

Our in vivo study showed that epithelial PBLD deficiency impairs intestinal barrier function by decreasing TJ proteins; we therefore explored the potential protective effect of PBLD on intestinal epithelial barrier function in vitro using Caco2 cells transfected with PBLD-expressing lentivirus, compared with a control lentivirus vector. The transfection efficiency was confirmed by qRT-PCR (Fig. 4a). In the absence of TNF-α/IFN-γ, the TEER values between Caco2 monolayers overexpressing PBLD and control cell monolayers were comparable, indicating similar barrier function (Fig. 4b). In the presence of TNF-α/IFN-γ, PBLD overexpression resulted in a smaller reduction in TEER values, indicating a protective effect against TNF-α/IFN-γ-induced impairments in intestinal barrier function (Fig. 4b). In the FITC-dextran flux assay, very little FITC-dextran passed through the Caco2 monolayers overexpressing PBLD, regardless of whether LPS were present or not, indicating decreased permeability (Fig. 4c). Western blotting revealed that the expression of ZO-1, occludin, and claudin-1 was markedly increased in the Caco2 monolayer overexpressing PBLD compared the control cell monolayer (Fig. 4d); this result was confirmed by qRT-PCR and immunofluorescent staining (Fig. 4e, f). In control cell monolayer, TNF-α/IFN-γ induced a decrease in expression and disrupted location of ZO-1 and occludin, which was consistent with previous research^34–36^. Overexpression of PBLD in the Caco2 monolayer not only prevented reductions in TJ proteins, but also improved the location of TJ proteins, which was disrupted by TNF-α/IFN-γ induction (Fig. 4f, g). PBLD overexpression also appeared to inhibit the NF-κB activation, which is normally induced by TNF-α/IFN-γ (Fig. 4g and Supplementary Fig. 8). In summary, these results indicate that PBLD improves epithelial barrier function by increasing the expression of TJ proteins and maintaining their correct location.

### Epithelial PBLD deficiency results in greater recruitment of infiltrating immune cells in murine colitis

**Fig. 5:**
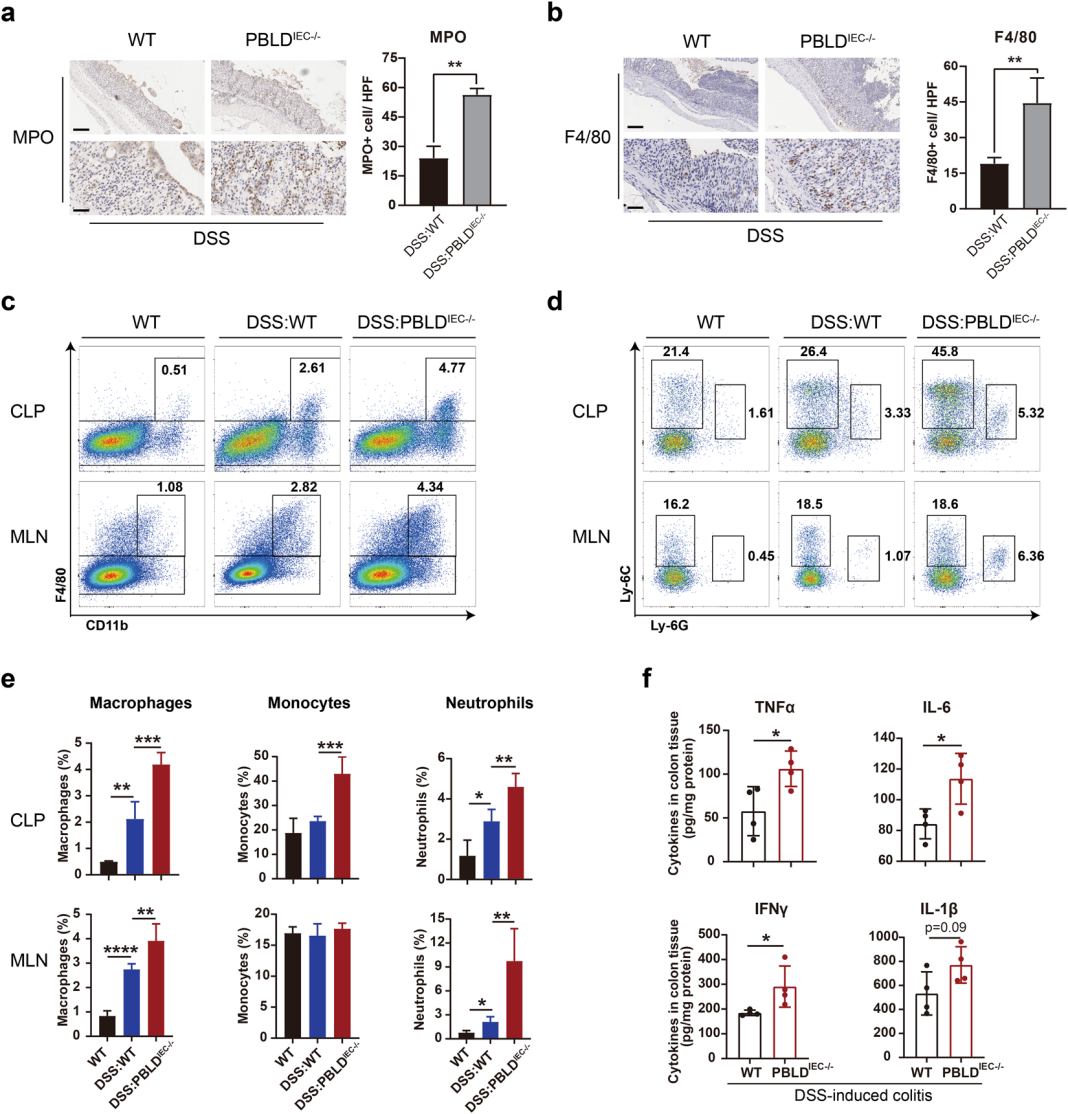
Epithelial PBLD deficiency recruits more immune cells infiltration in murine colitis. **a** Myeloperoxidase (MPO) and (**b**) F4/80-stained colonic tissue sections in mice. Images are representative from three to four mice in each group. Scale bars: 200 μm in upper in both (**a** and **b**), 50 μm in lower in both (**a** and **b**). **c** Cells isolated from colonic lamina propria or mesenteric lymph nodes were labeled with anti-F4/80 and anti-CD11b, **d** anti-Ly-6C and anti-Ly-6G, and then visualized via flow cytometry and **e** their corresponding proportions of macrophages, monocytes, and neutrophils in CLP or MLN. WT, *n* = 3; DSS:WT, *n* = 5; DSS:PBLD^IEC−/−^, *n* = 5. CLP colonic lamina propria, MLN mesenteric lymph node. **f** Enzyme-linked immunosorbent assay (ELISA) of whole-colon homogenates in mice to evaluate TNF-α, IL-6, IFN-γ, and IL-1β production (*n* = 4). Data are mean ± standard deviation (SD). *p < 0.05.

To evaluate the infiltration of immune cells in the colon, we performed MPO and F4/80 staining to detect neutrophils and macrophages, respectively, in colonic tissue sections from mice with DSS-induced colitis. We found that these cell types were markedly increased in colon tissue from PBLD^IEC−/−^ mice compared with WT mice (Fig. 5a, b). Epithelial PBLD deficiency was associated with increased macrophage, monocyte, neutrophil, and CD4^+^ T cell infiltration in the CLP and MLN of mice with DSS-induced colitis, as demonstrated in our flow cytometry assays (Fig. 5c–e and Supplementary Fig. 9). Using ELISA, we also found the expression levels of the pro-inflammatory cytokines TNF-α, IL-6, IFN-γ, and IL-1β were increased in PBLD^IEC−/−^ mice versus WT mice with DSS-induced colitis (Fig. 5f). In addition, epithelial PBLD deficiency in TNBS-induced colitis was associated with decreased CD4^+^ Foxp3+ cells (a marker of T regulatory cells) and increased IL-17A+ cells (Supplementary Fig. 10a). The expression levels of TNF-α and IL-6 were also increased in PBLD^IEC−/−^ mice compared with WT mice with TNBS-induced colitis (Supplementary Fig. 10b). Collectively, these results suggest that PBLD deficiency in IECs may lead to greater immune cell infiltration in experimental colitis.

### Loss of PBLD expression in IECs leads to excessive NF-κB activation in DSS-induced colitis

**Fig. 6:**
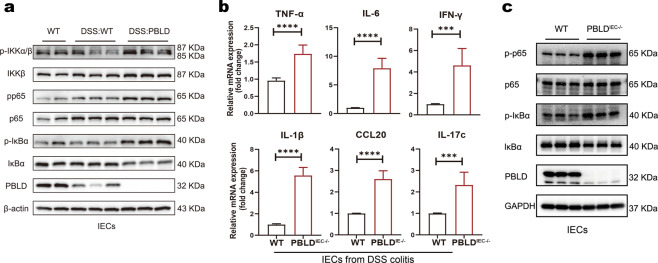
Loss of PBLD in IECs leads to excessive NF-κB activation in DSS-induced colitis. **a** Western blot analysis of proteins in IECs isolated from WT mice and PBLD^IEC−/−^ mice treated with DSS for 5 days followed by 3 days regular drinking water. **b** qRT-PCR analysis of relative mRNA levels of genes in IECs isolated from WT mice and PBLD^IEC−/−^ mice with DSS-induced colitis (*n* = 6). Data are mean ± standard deviation (SD). ***p < 0.001. **c** Immunoblotting analysis of proteins in primary IECs isolated from WT and PBLD^IEC−/−^ mice after TNF-α treatment for 1 h.

PBLD may regulate NF-κB signaling^17^, which controls the expression of inflammatory mediators and play a crucial role in IBD^14^. We found that expression of two NF-κB signaling key molecules, p-p65 and p-IκBα, was markedly increased in IECs isolated from PBLD^IEC−/−^ mice compared with those from WT mice in DSS-induced colitis (Fig. 6a and Supplementary Fig. 11), indicating epithelial PBLD deficiency may enhance NF-κB activity in IECs. The expression of inflammatory mediators (TNF-α, IL-6, IFN-γ, IL-1β, CCL20, and IL-17c) were significantly increased in IECs isolated from the PBLD^IEC−/−^ compared with the WT mice (Fig. 6b). We also found that when NF-κB signaling was activated using TNF-α, NF-κB activation was significantly enhanced in IECs isolated from PBLD-deficient mice compared with WT mice (Fig. 6c and Supplementary Fig. 12). Collectively, these results demonstrate that loss of PBLD in IECs leads to excessive NF-κB activation in DSS-induced colitis.

### PBLD inhibits the TNF-α-induced inflammatory response in IECs by suppressing NF-κB activation

**Fig. 7:**
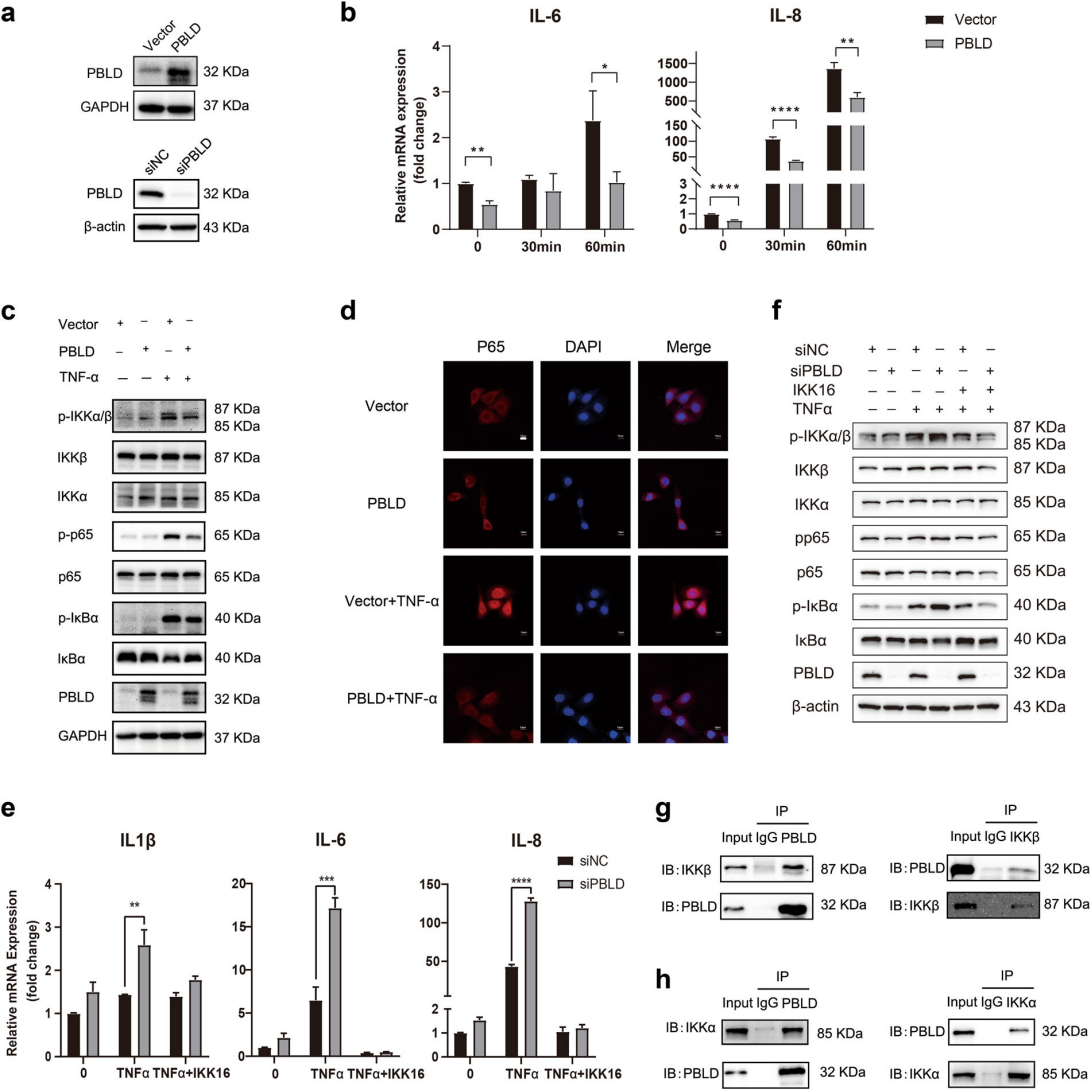
PBLD suppresses TNF-α-induced inflammatory responses in IECs by inhibiting NF-κB activation. **a** Western blot showing the expression of PBLD in a fetal human cell line (FHC) transfected with PBLD-expressing lentivirus (FHC-PBLD) or control lentivirus vector (FHC-vector) (upper) and siNC or siPBLD (lower). **b** qRT-PCR results showing the production of inflammatory mediators in FHC-vector and FHC-PBLD after TNF-α (10 ng/mL) treatment for the indicated amount of time. Data are mean±standard deviation (SD). **p < 0.01. **c** Western blot analysis of proteins in FHC-vector and FHC-PBLD cells after TNF-α (10 ng/mL) treatment for 1 hour. **d** Immunofluorescent staining for p65 in FHC-vector and FHC-PBLD cells after TNF-α (10 ng/mL) treatment for 1 hour. Scale bars: 10 μm. **e** qRT-PCR analysis of inflammatory mediators production and (**f**) immunoblot analysis of NF-κB signal pathway in FHC cells transfected with siNC or siPBLD, treated with 5 µM IKK-16 for 2 hours, followed by TNF-α (10 ng/mL) treatment for 1 h. **g** Immunoblot analysis of IKKβ and PBLD expression in a co-immunoprecipitation assay with anti-PBLD (left) or anti-IKKβ (right) antibodies in FHC-PBLD cells. **h** Immunoblot analysis of IKKα and PBLD expression in a co-immunoprecipitation assay with anti-PBLD (left) or anti-IKKα (right) antibodies in FHC-PBLD cells.

To further investigate the potential regulation of NF-κB signaling by PBLD, we evaluated the effect of PBLD on inflammatory responses in IECs stimulated with TNF-α. We found that TNF-α induction resulted in increased expression of inflammatory mediators (IL-8, IL-6, IL-1β, and TNF-α), secreted by IECs. The production of inflammatory mediators in FHC and HT29 cells overexpressing PBLD was significantly decreased compared with control cells (Fig. 7a, b and Supplementary Fig. 13a, b). TNF-α induction resulted in the increased expression of p-p65 and p-IκBα, but this effect was attenuated by PBLD overexpression in FHC cells (Fig. 7c and Supplementary Fig. 14). We also found that, in FHC and HT29 cells overexpressing PBLD, TNF-α-induced translocation of p65 from the cytoplasm into the nucleus was blocked (Fig. 7d and Supplementary Fig. 13c). In contrast, PBLD knockdown in FHC cells resulted in significantly increased production of inflammatory mediators (IL-1β, IL-6, and IL-8) and enhanced NF-κB activation compared with control cells; this effect was abolished by the IKK inhibitor IKK-16 (Fig. 7e, f). Furthermore, co-immunoprecipitation assays confirmed an interaction between PBLD and both IKKα and IKKβ in FHC cells, and vice versa. Collectively, these results indicate that PBLD is able to bind to IKKα and IKKβ to inhibit NF-κB activation, suppressing TNF-α-induced inflammatory responses in IECs.

## Discussion

PBLD, also termed MAWBP, was first identified from a human liver cDNA library by using the yeast two hybrid system^37^. Recent studies have indicated that PBLD is markedly decreased in gastric cancer, breast cancer, and liver cancer, and suppresses the development and progression of these cancers^15–18,38,39^. Although the role of PBLD in cancer is relatively clear, its role in UC remains unknown. In the previous study, we demonstrated that PBLD was significantly decreased in UC and negatively correlated with the disease severity, suggesting PBLD might serve as a potential biomarker for monitoring disease progression^19^. In the present study, we demonstrated that PBLD plays a protective role in the pathogenesis of UC by using genetically engineered mice and chemical-induced mouse colitis model.

The intestinal epithelium both protects mucosal immune cells from pathogens, and recruits immune cells to sites of inflammation by secreting inflammatory mediators^40^. Intestinal barrier function and integrity, and interactions between IECs and immune cells, thus contribute to intestinal homeostasis; dysregulated immune responses thus result in disruption of intestinal homeostasis, leading to sustained and uncontrolled inflammation^7,41^. In our in vivo experiments, we found that the loss of PBLD in IECs exacerbated intestinal inflammation in DSS-induced colitis, and resulted in significant intestinal barrier functional defects and extensive immune cell infiltration. We also found that the overexpression of PBLD in IECs resulted in reduced production of inflammatory mediators and improved intestinal epithelial barrier function under inflammatory conditions. PBLD may therefore be an important regulator of intestinal barrier function and immunomodulatory activity in IECs in UC.

Intestinal barrier function refers to the ability of the intestine to restrict microbiota and absorb nutrients from the gut lumen. TJ proteins, including ZO-1, occludin, and claudins, regulate intestinal permeability^42^. Previous studies have reported alteration of TJ structure and reduction of TJ proteins, such as ZO-1 and occludin resulted in intestinal barrier functional defects in patients with UC^32,43–45^. In this study, the levels of TJ proteins were lower in inflamed compared with non-inflamed colon tissue samples taken from UC patients, and expression of TJ proteins was positively correlated with PBLD expression in these tissue samples. Furthermore, PBLD overexpression in Caco2 cell monolayers resulted in the decreased epithelial permeability and improved expression of TJ proteins in inflammation, while PBLD deficiency in IECs resulted in increased intestinal permeability and decreased TJ proteins expression in DSS-induced colitis. Similarly to previous studies demonstrating that PBLD increases the expression of E-cadherin^17,38^, which contributes to adherence between cells, our study indicates that PBLD increases the expression of TJ proteins, thus reducing permeability of the intestinal barrier. This is consistent with previous in vitro and in vivo studies, which reported that increased expression of TJ proteins may improve intestinal epithelial barrier function^46,47^; however, the mechanism underlying PBLD regulation of TJ protein expression remains unknown. The location of TJ proteins is also known to affect intestinal permeability; pro-inflammatory cytokines (such as TNF-α, IL-1β, and IFN-γ) increase the expression of MLCK, which results in the disruption of TJ protein location and thus increased intestinal permeability^48–50^. This process is blocked by NF-κB inhibition, indicating that TNF-α induced MLCK expression requires NF-κB activation^51,52^. In a Caco2 cell monolayer, we found that the PBLD overexpression inhibited NF-κB activation and improved the disruption of TJ induced by IFN-γ/TNF-α. Collectively, we speculated that PBLD might improve epithelial intestinal barrier function in the colonic inflammation by increasing the expression of TJ proteins and regulating the NF-κB/MLCK axis. However, more studies about how PBLD regulates TJ expression are needed to perform in the future. Therefore, the role of PBLD in improving intestinal barrier function may provide a potential approach for repairing intestinal barrier function defects in patients with UC.

In response to microbial stimuli or cytokines, IECs can secrete chemokines and cytokines to coordinate appropriate immune responses in healthy individuals^6,11^. The secretion of inflammatory mediators from IECs is mainly regulated by the NF-κB signaling pathway^14^; however, the role of NF-κB in IECs are currently not well characterized. Some previous studies have demonstrated that NF-κB signaling inhibition by epithelial knockouts of IKKα, IKKβ, and/or NEMO resulted in exacerbated DSS-induced colitis or spontaneous colitis, owing to extensive apoptosis of IECs^53,54^. Another study indicated that persistent NF-κB activation in IECs led to increased secretion of various chemokines and cytokines, including TNF-α, CCL20, CCL2, and macrophage colony stimulating factor, resulting in increased immune cell infiltration in the gut, and mucosal immune system overreaction in response to environmental stimuli^55^. Complete ablation or persistent activation of NF-κB signaling in IECs disrupts their homeostatic functions and leads to intestinal inflammation. Thus, maintaining NF-κB activity within an appropriate range may help to defend against intestinal inflammation^56^. In the present study, we found that PBLD deficiency in IECs led to excessive NF-κB activation in mice with DSS-induced colitis, accompanied by increased chemokine and cytokine secretion and greater immune cell infiltration in the colon. Conversely, in vitro PBLD overexpression in IECs resulted in decreased chemokine and cytokine secretion by attenuating NF-κB activity. Therefore, PBLD may regulate NF-κB activation in the context of inflammation. We also found that an IKK inhibitor rescued increases in inflammatory mediator production and excessive NF-κB activation in PBLD knockdown FHC cells treated with TNF-α. Furthermore, a co-immunoprecipitation assay revealed that PBLD was able to bind to IKKα and IKKβ. Recent studies have reported that proteins that bind to IKKα and IKKβ can inhibit the NF-κB activation by suppressing IKK activity^57,58^. Our results suggest that the interaction between PBLD and IKKα/IKKβ may suppress IKK activity, thus attenuating NF-κB activation. PBLD may therefore decrease the secretion by attenuating NF-κB signaling, which may represent a potential anti-inflammatory approach for UC treatment.

**Fig. 8:**
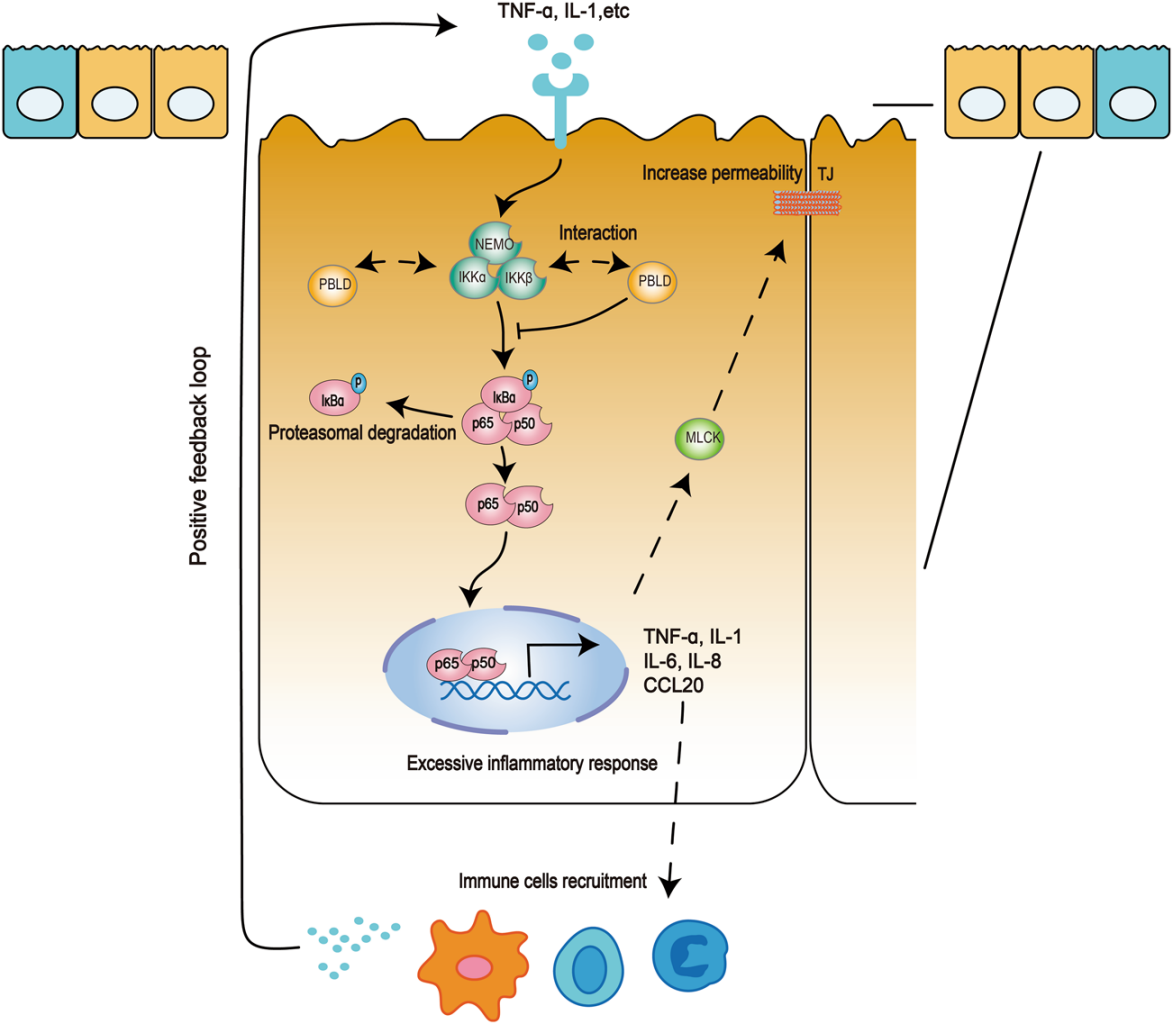
Model of how PBLD regulates NF-κB signaling pathway in the pathogenesis of UC. In response to microbial stimuli or cytokines, NF-κB is activated in intestinal epithelial cells (IECs), which may impair epithelial barrier function and increase chemokine and cytokine production, resulting in the infiltration of more immune cells. When external stimuli persist, the recruited immune cells may produce more cytokines, leading to uncontrolled inflammation. PBLD may interact with IKKα and IKKβ to prevent excessive inflammatory responses in IECs, thus attenuating intestinal mucosal inflammation and improving epithelial barrier function.

In summary, our study demonstrated that PBLD was downregulated in UC patients and a mouse model of DSS-induced colitis. Our results indicate that epithelial PBLD prevents intestinal mucosal inflammation and improves intestinal barrier function by inhibiting NF-κB signaling (Fig. 8). PBLD may therefore regulate NF-κB activity and cytokine/chemokine signaling in UC, and modulating PBLD expression may represent a novel approach to improve treatment for patients with UC.

## Supplementary information

Supplementary Information
