# Supplementary material for: Epithelial PBLD attenuates intestinal inflammatory response and improves intestinal barrier function by inhibiting NF-κB signaling
Source: Cell Death Dis. 2021 May 31;12(6):563. doi: 10.1038/s41419-021-03843-0 (PMC8166876; doi:10.1038/s41419-021-03843-0)
Supplement: Supplementary file 1 — Supplementary Information [file 41419_2021_3843_MOESM1_ESM.doc]

**Supplementary Information**

**Epithelial PBLD attenuates intestinal inflammatory response and improves intestinal barrier function by inhibiting NF-κB signaling**

Shengbo Chen^1^, Hongbin Liu^1^, Zhijun Li^1^, Jingyi Tang^1^, Bing Huang^1^, Fachao Zhi*^1^, Xinmei Zhao*^1^.

1. Guangdong Provincial Key Laboratory of Gastroenterology, Institute of Gastroenterology of Guangdong Province, Department of Gastroenterology, Nanfang Hospital, Southern Medical University, Guangzhou 510515, China

*Corresponding authors: Xinmei Zhao (xmzhao914@163.com), Fachao Zhi (zhifc41532@163.com)

**Supplementary Figures:**

Supplementary Figure 1- Supplementary Figure 14

**Supplementary Tables:**

Supplementary Table1-Supplementary Table6

**Supplementary Figures**


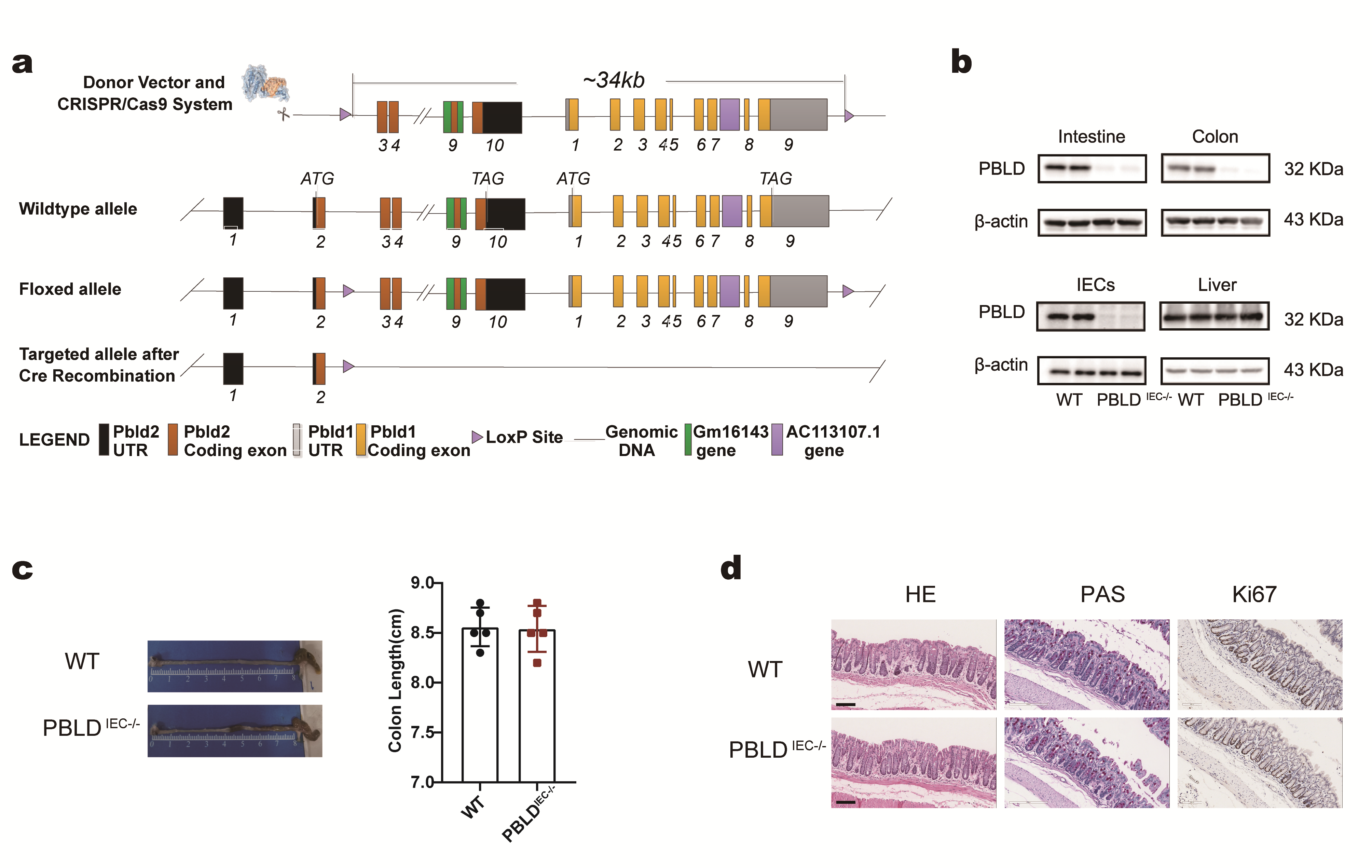


**Supplementary Figure 1. Epithelial phenazine biosynthesis-like domain-containing protein (PBLD) knockout mice have normal colonic function under baseline conditions**

**a** Generation of epithelial PBLD deficient mice. **b** Western blot analysis showing expression of PBLD in small intestine tissue, colon tissue, isolated intestinal epithelial cells (IECs) and liver tissue of epithelial PBLD deficient mice. β-actin was used as an internal control. **c** Colon length of wild-type (WT) mice and PBLD^IEC-/-^ mice (n = 5). **d** hematoxylin-eosin (HE) staining (left), periodic acid-Schiff (PAS) staining (middle) and ki67 labeling (right) of colon sections from WT mice and PBLD^IEC-/-^ mice. Scale bar: 100 µm.


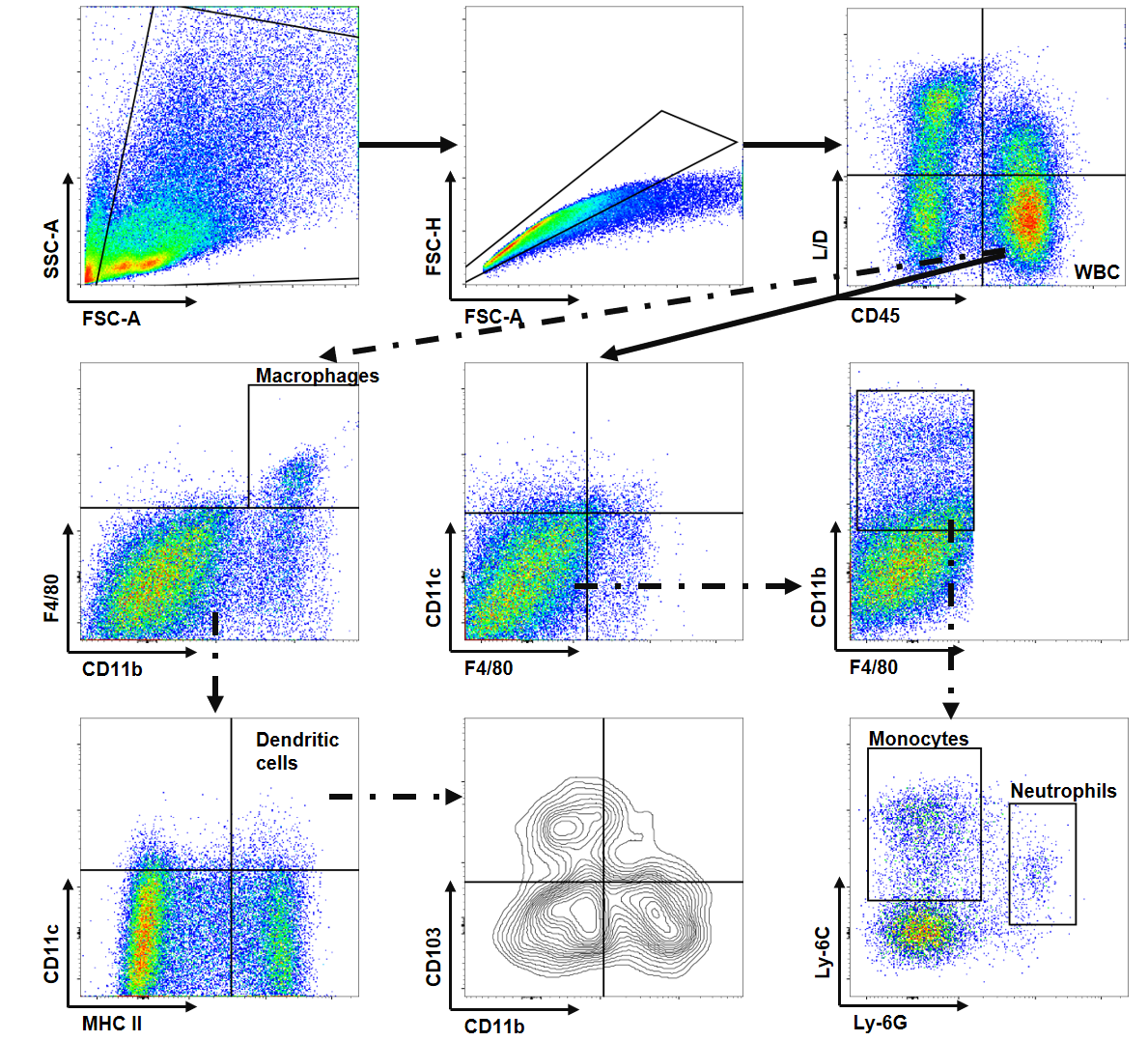


**Supplementary Fig. 2 Gating strategy for flow cytometry analysis of innate immune cells infiltration in the colonic lamina propria (CLP) and mesenteric lymph nodes (MLN).**


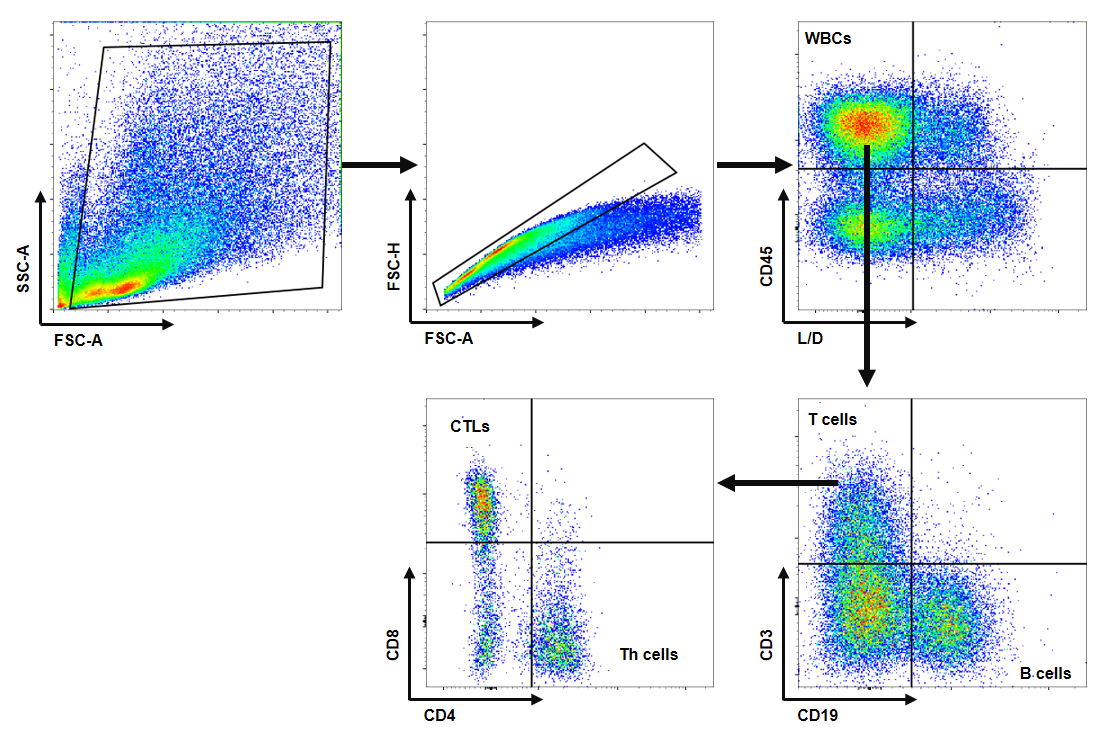


**Supplementary Fig.3 Gating strategy for flow cytometry analysis of adaptive immune cells infiltration in the colonic lamina propria (CLP) and mesenteric lymph nodes (MLN).**


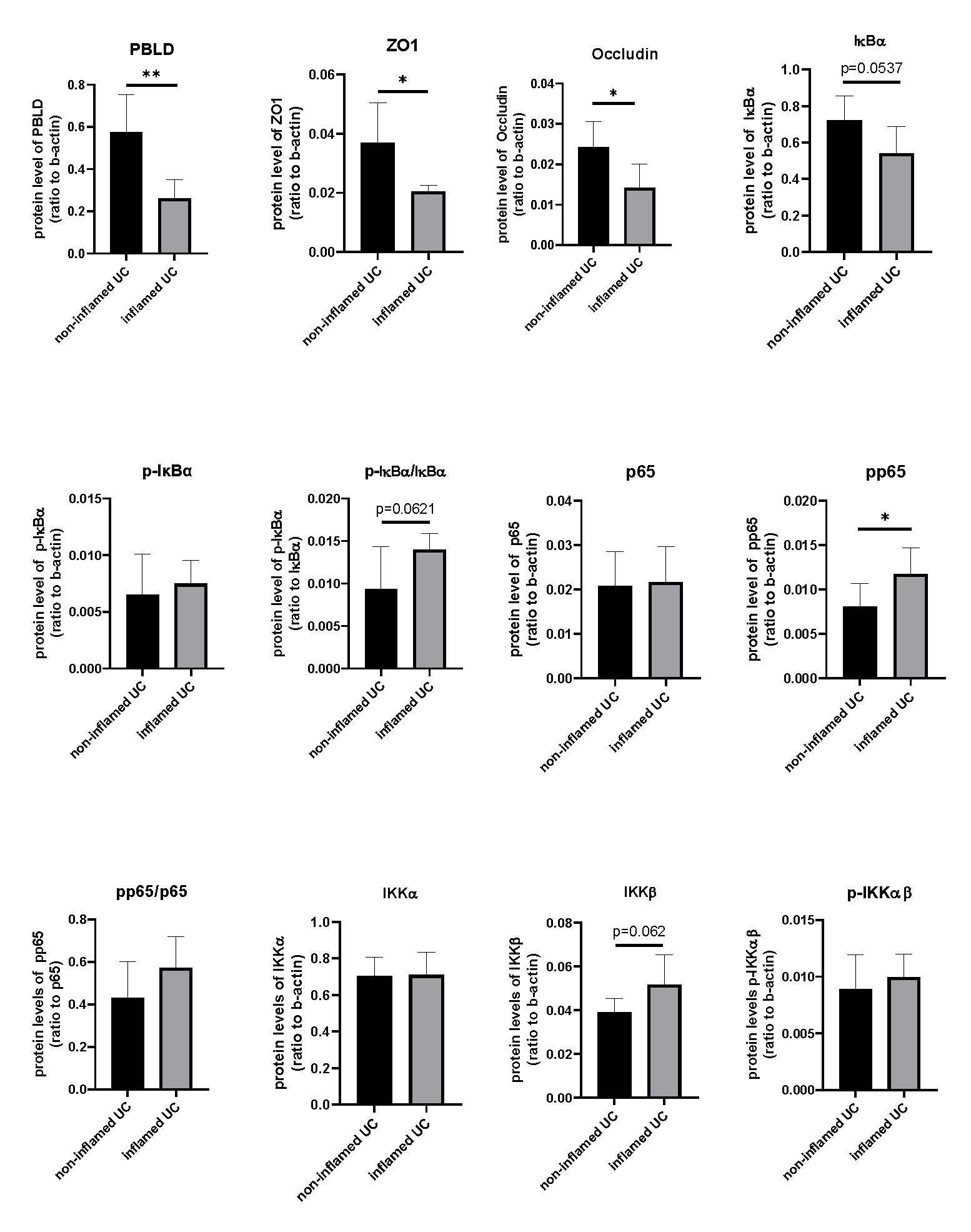


**Supplementary Fig.4 Densitometry quantification showing the reduction of PBLD and tight junction (TJ) proteins, and nuclear factor (NF)-κB activation in inflamed colonic tissue from patients with UC compared with paired non-inflamed colonic tissue samples.**


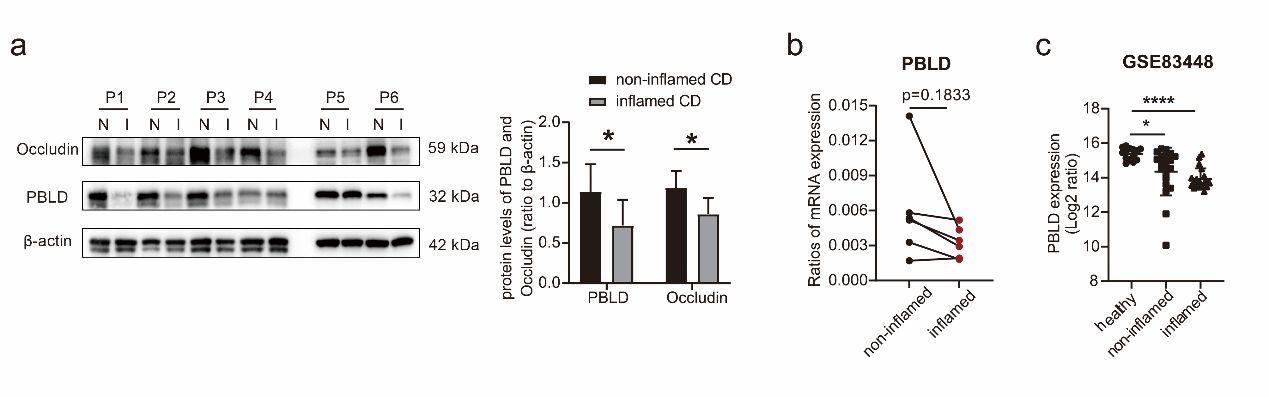


**Supplementary Fig.5 PBLD is decreased in patients with Crohn’s disease.**

**a** Expression of PBLD and occludin was evaluated by immunoblotting in paired inflamed and non-inflamed colonic tissue samples from people with CD (n = 6). β-actin was used as an internal control. N, Normal colon tissue; I, inflamed colon tissue. **b** Expression of PBLD mRNA was analyzed by qRT-PCR in paired inflamed and non-inflamed colonic tissue samples from people with CD (n = 6). GAPDH was used as an internal control. ***p* < 0.01. **c** Expression of PBLD mRNA was assessed using a published dataset (GSE83448; N = 53; healthy control, n = 14; non-inflamed, n = 20; inflamed, n = 19) **** *p*< 0.0001.

**
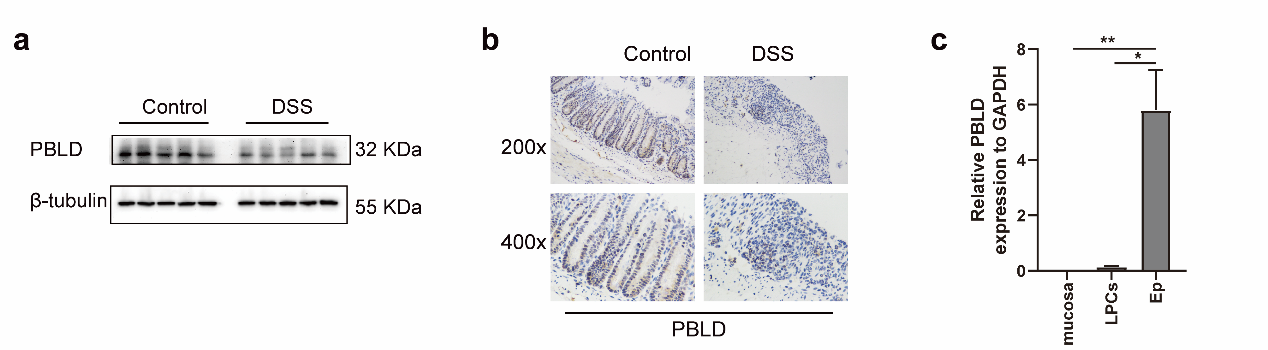
**

**Supplementary Fig.6 PBLD is decreased in DSS-induced colitis.**

**a** Western blot showing PBLD expression in colonic tissue of mice that underwent 5 days of DSS treatment, followed by 3 days of regular drinking water (n = 5). β-tubulin served as an internal control. **b** Immunohistochemical staining of PBLD in colonic sections from normal control and DSS-induced colitis. **c** qRT-PCR analysis showing the abundance of PBLD in the mucosa, lamina propria cells (LPCs) and intestinal epithelial cells (IECs) (n = 3–4). GAPDH mRNA served as an internal control. Data are mean ± standard deviation (SD). **p* < 0.05, ***p* < 0.01.


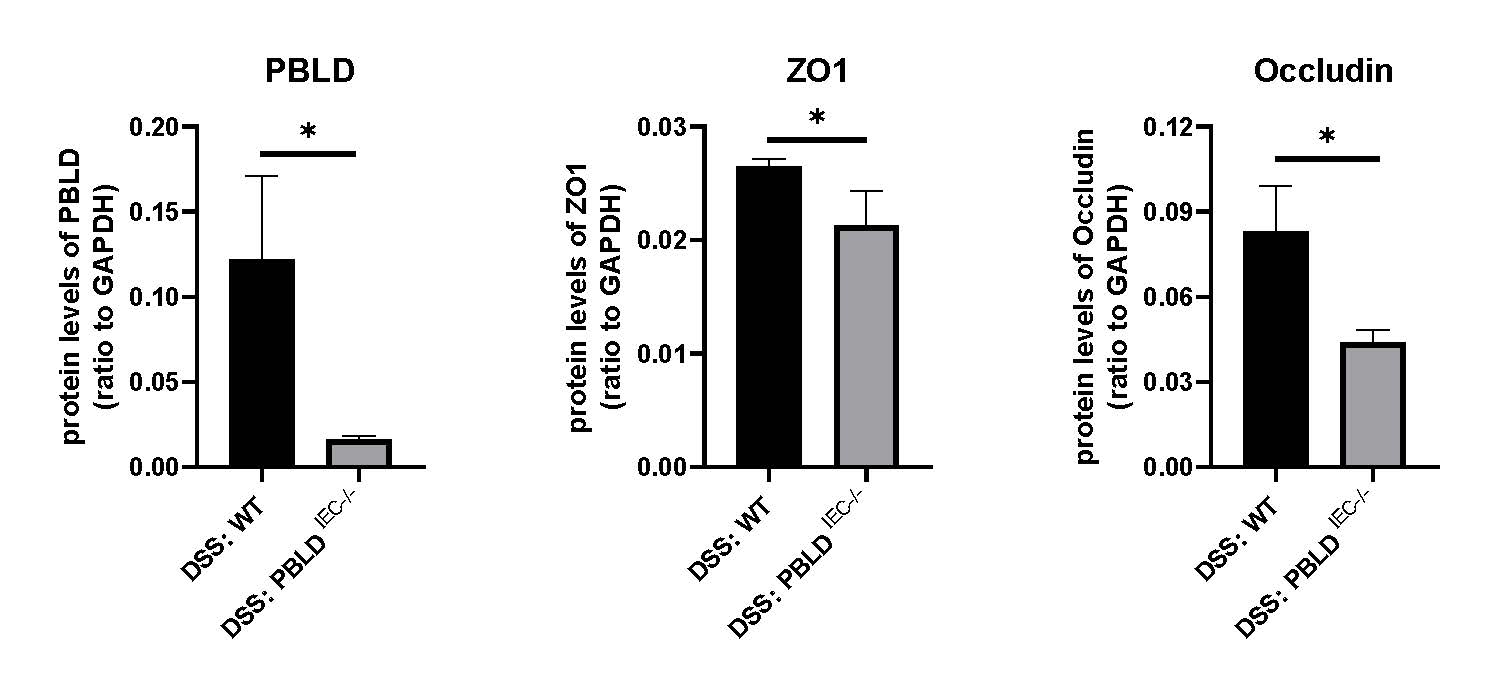


**Supplementary Fig.7 Densitometry quantification showing decreased ZO-1 and occludin in colonic tissue of PBLD^IEC-/-^ mice compared with WT mice with DSS-induced colitis.**


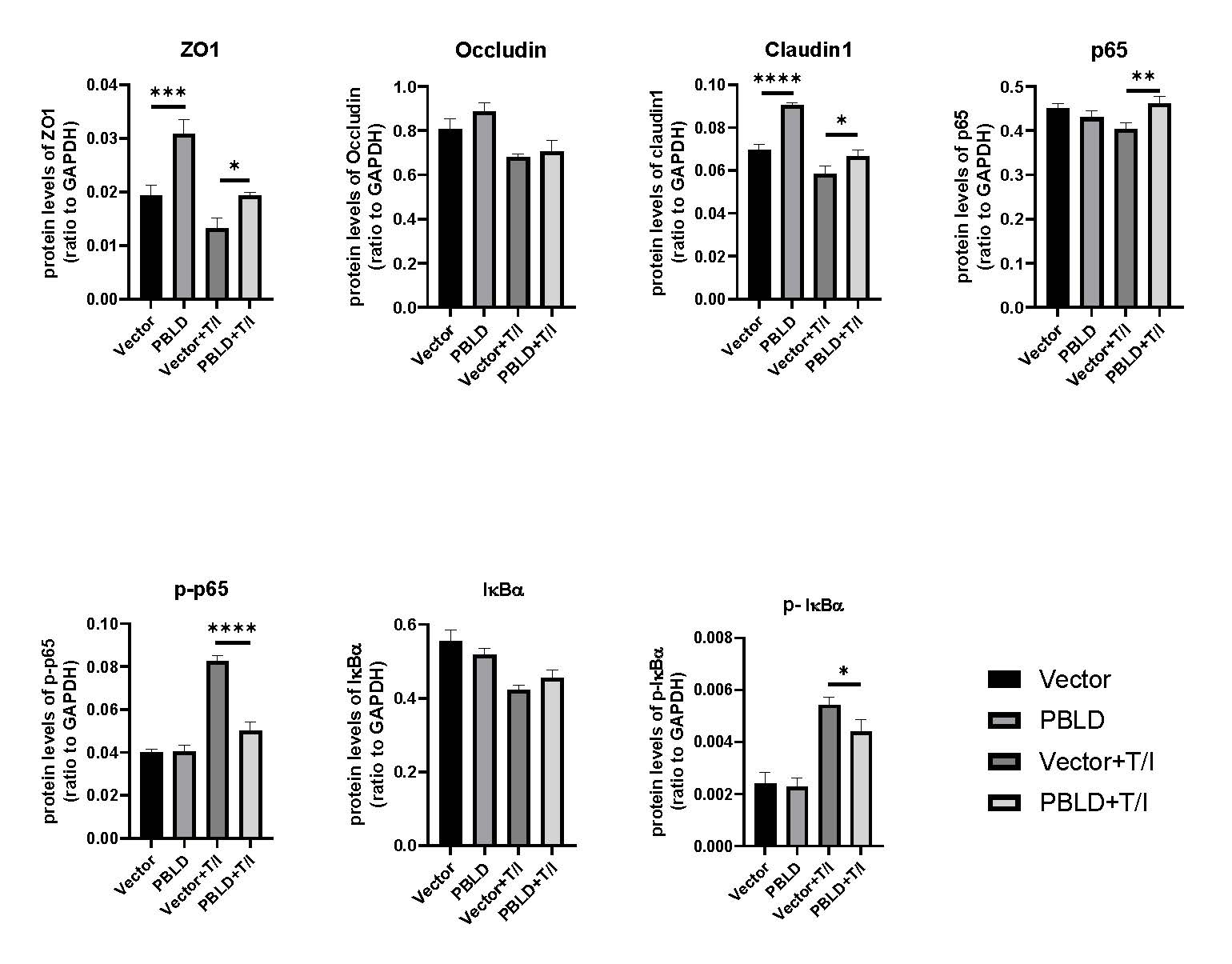


**Supplementary Fig.8 Densitometry quantification showing PBLD increases tight junction (TJ) protein expression and inhibits NF-κB activation induced by TNF-α/ IFN-γ in a Caco2 cell monolayer.**


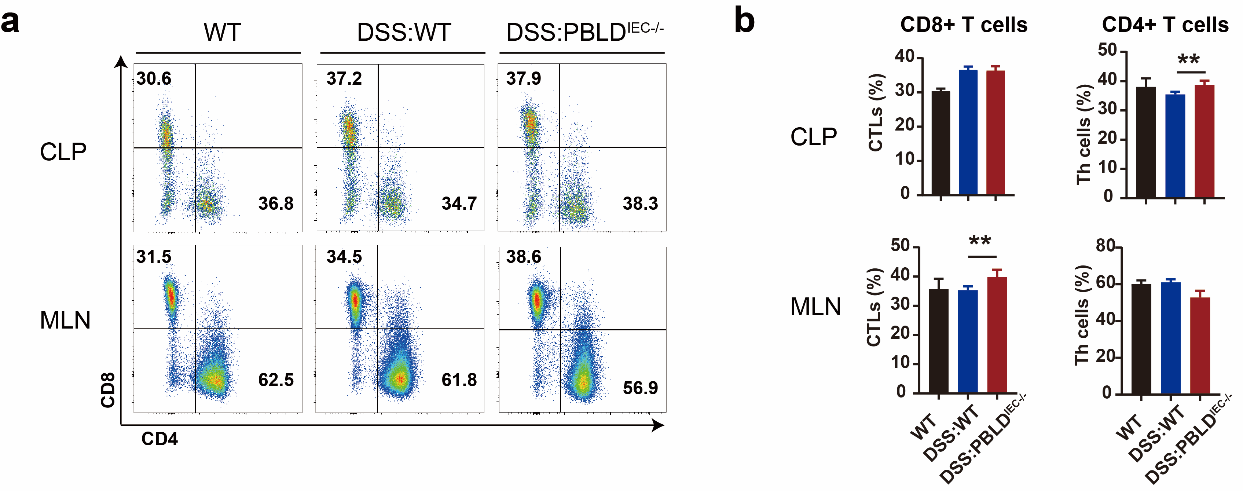


**Supplementary Fig.9 PBLD deficiency increases T cells in DSS-induced colitis.**

**a-b** The indicated mice were treated with DSS for 5 days, the proportions of cytotoxic T lymphocytes (CLTs) and T helper (Th) cells in the colonic lamina propria or mesenteric lymph nodes were analyzed by flow cytometry. WT, n=3; DSS:WT, n=5; DSS:PBLDIEC-/-, n=5. CLP, Colonic lamina propria; MLN, Mesenteric lymph node.


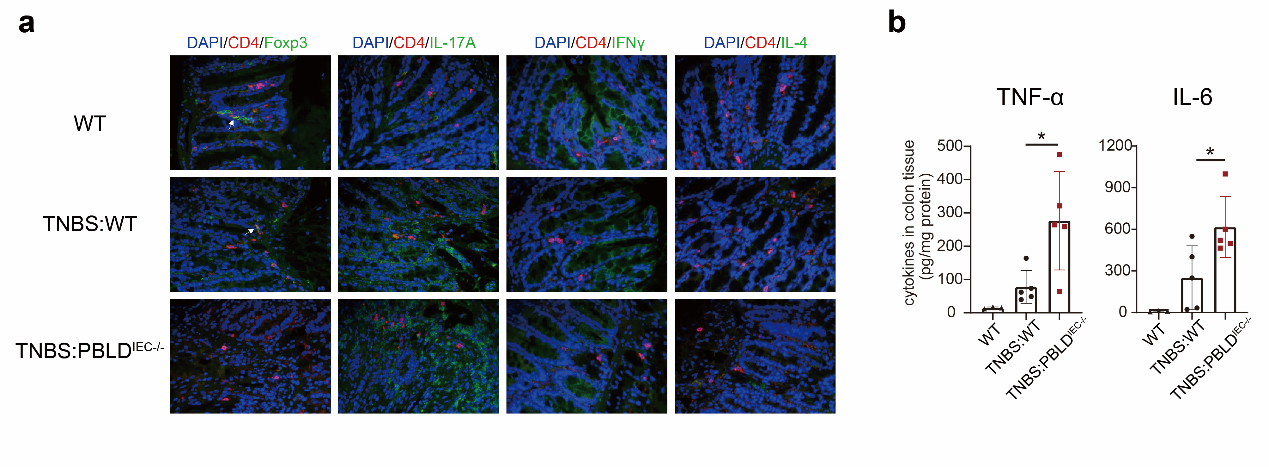


**Supplementary Fig.10 The effect of PBLD on T cells in TNBS-induced colitis in mice.**

**a.** Representative immunofluorescence staining for cell determinant (CD)4, Foxp3, interleukin (IL)-17A, interferon (IFN)-γ and IL-4 in the colonic section of mice (at 400 x magnification). **b.** The expression of TNF-α and IL-6 in the colon tissue of indicated mice was evaluated by ELISA (n=3-5).


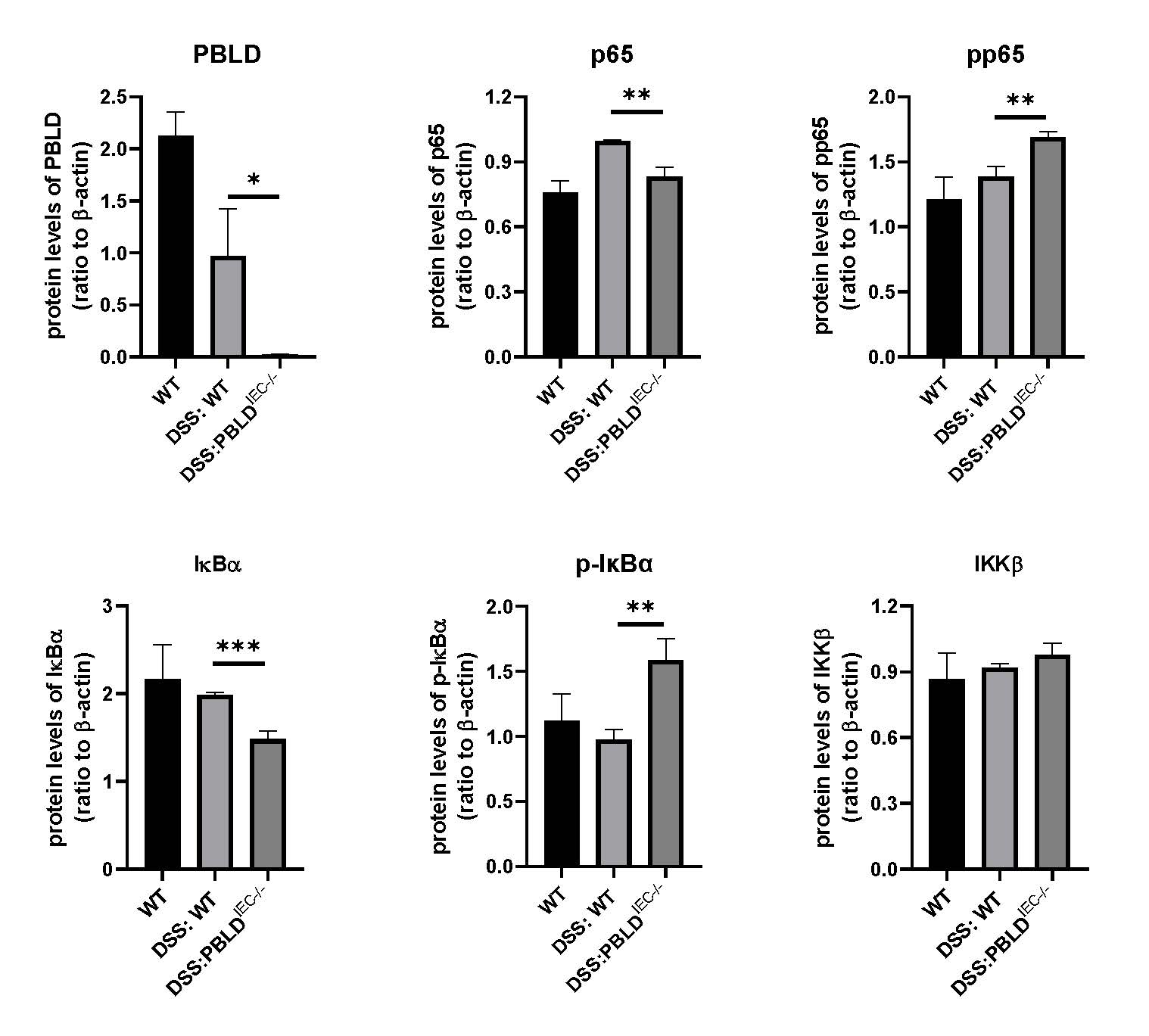


**Supplementary Fig.11 Densitometry quantification showing that epithelial PBLD deficiency enhances NF-κB activation in DSS-induced colitis in mice.**

**
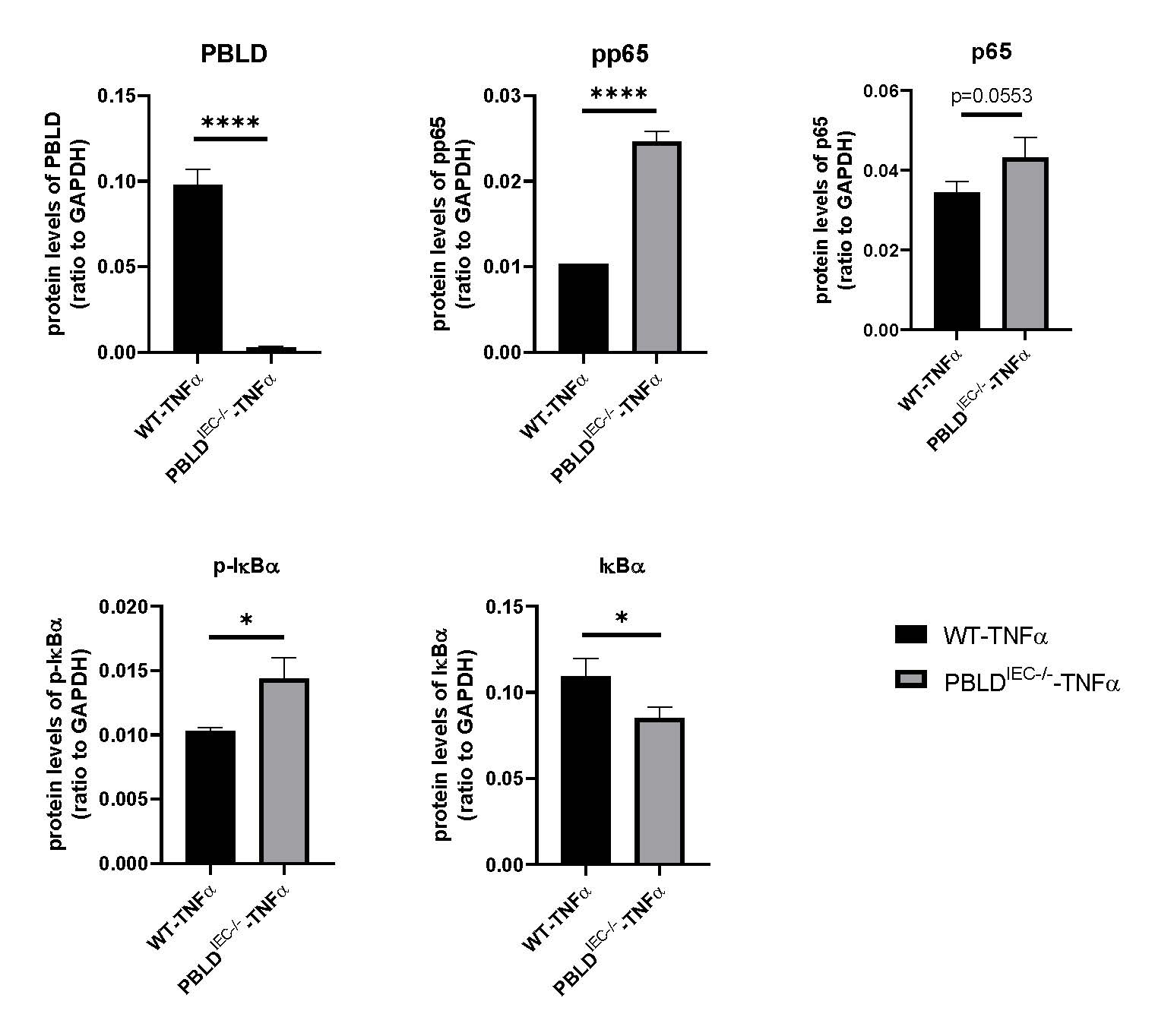
**

**Supplementary Fig.12 Densitometry quantification showing that epithelial PBLD deficiency enhances NF-κB activation induced by TNF-α.**

**_
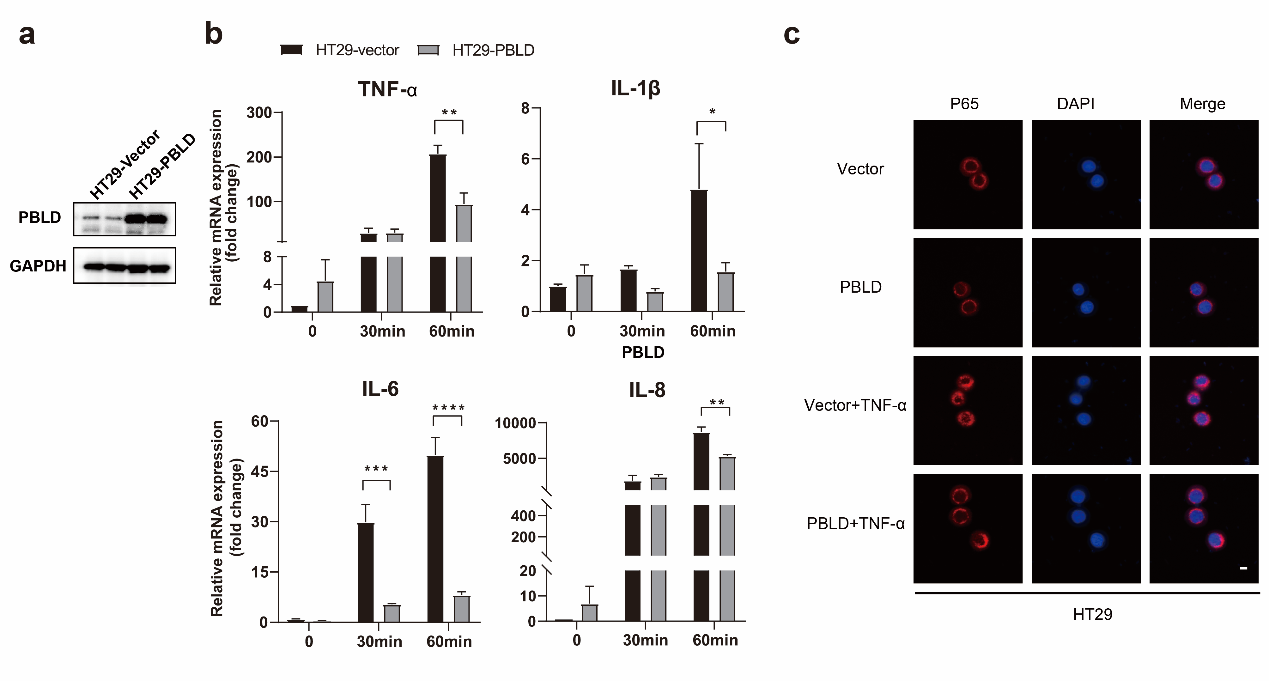
_**

**Supplementary Fig.13 PBLD inhibits TNF-α-induced inflammatory response in HT29 cells via the suppression of NF-κB activation**

**a** HT29 cells were transfected with PBLD-expressing lentivirus or a control lentiviral vector; the expression of PBLD was then evaluated using a western blot. **b** The production of inflammatory mediators in HT29-vector and PBLD-overexpressing HT29 cells (HT29-PBLD) after TNF-α (10 ng/mL) treatment was assessed by qRT-PCR. Data are mean ± standard deviation (SD). ***p* < 0.01. **c** Immunofluorescent staining for p65 in HT29-vector and HT29-PBLD cells after TNF-α (10 ng/mL) treatment for 1 hour. Scale bars: 10 μm.

**
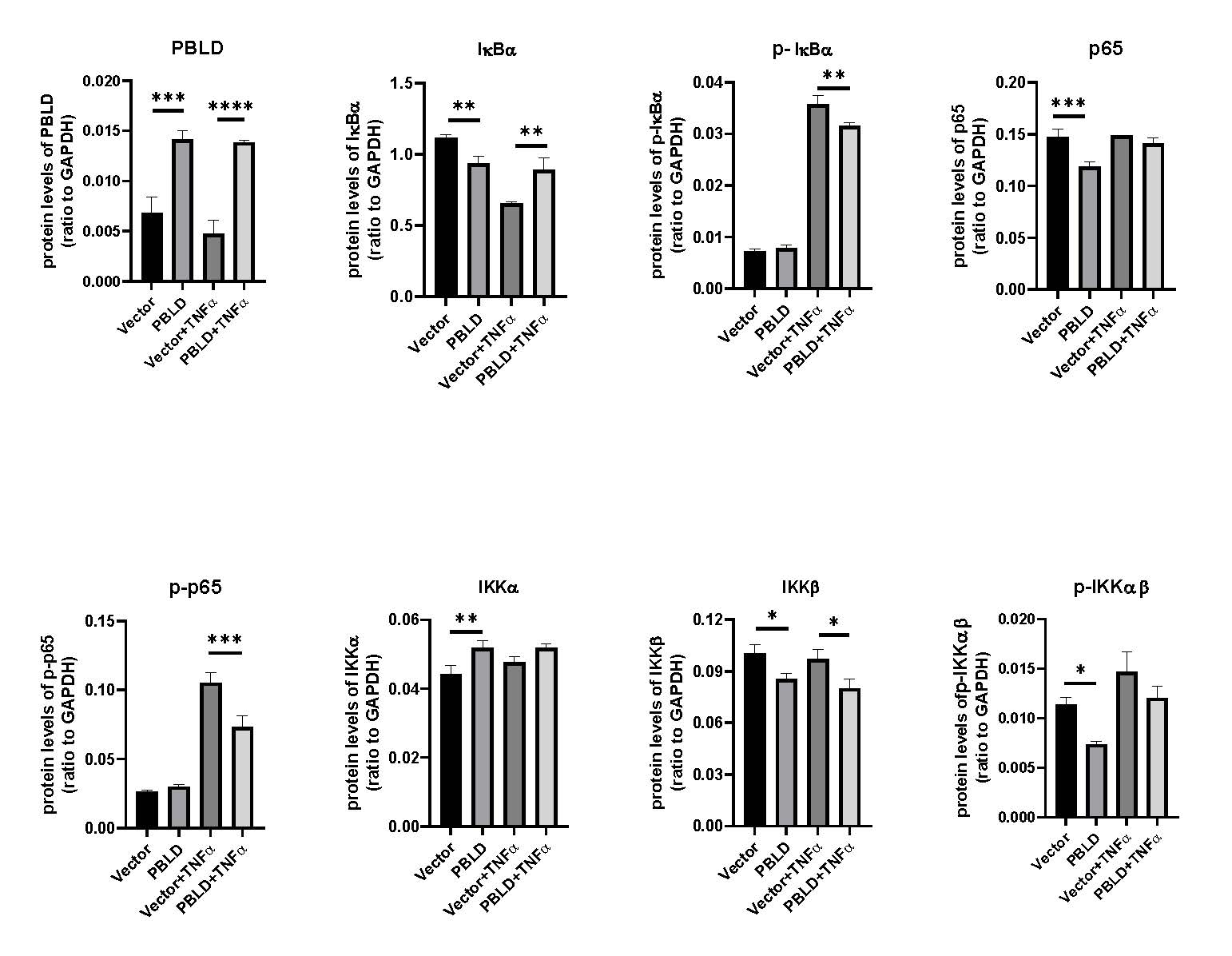
**

**Supplementary Fig.14 Densitometry quantification showing that PBLD inhibits NF-κB activation induced by TNF-α in FHC cell line.**

**Supplementary Tables**

**Supplementary Table 1 The characteristics of patients with ulcerative colitis**

| **Characteristics** | **UC (n=9)** |
| --- | --- |
| **Gender** |  |
| Male | 6 |
| Female | 3 |
| **Age (years ± SD)** | 45 ± 16.33 |
| **Disease duration (months ± SD)** | 43.56 ± 40.63 |
| **Extent of disease** |  |
| Extensive colitis (E3) | 3 |
| Left-sided colitis (E2) | 1 |
| Proctitis (E1) | 5 |
| **Severity** |  |
| Remission (S0) | 0 |
| Mild UC (S1) | 1 |
| Moderate UC (S2) | 6 |
| Severe UC (S3) | 2 |
| **Treatment** |  |
| TNF antagonist use | 0 |
| Aminosalicylates | 7 |
| Immune modulator use | 0 |
| Corticosteroids | 0 |
| None | 2 |

**Supplementary Table 2 The characteristics of patients with Crohn’s disease**

| **Characteristics** | **CD (n=6)** |
| --- | --- |
| **Gender** |  |
| Male | 3 |
| Female | 3 |
| **Age at diagnosis** |  |
| <16 years (A1) | 2 |
| 17-40 years (A2) | 4 |
| >40 years (A3) | 0 |
| **Disease location** |  |
| Ileal disease (L1) | 2 |
| Colonic disease (L2) | 2 |
| Ileocolonic disease (L3) | 2 |
| Upper-isolated gastrointestinal disease (L4) | 0 |
| **Disease behaviour** |  |
| Non‐stricturing and non‐penetrating (B1) | 6 |
| Stricturing (B2) | 0 |
| Penetrating (B3) | 0 |
| Perianal disease (P) | 0 |
| **Treatment** |  |
| TNF antagonist use | 2 |
| Aminosalicylates | 2 |
| Immune modulator use | 3 |
| Corticosteroids | 0 |
| None | 2 |

**Supplementary Table 3 The antibodies and reagents used in this study**

| **REAGENTS or ANTIBODIES** | **SOURCE** | **IDENTIFIER** |
| --- | --- | --- |
| Anti-PBLD | Proteintech | 27891-1-AP |
| Anti-PBLD | Sigma | HPA038036 |
| Anti-ZO1 | Proteintech | 21773-1-AP |
| Anti-Occludin | Proteintech | 27260-1-AP |
| Anti-Claudin1 | Proteintech | 13050-1-AP |
| Anti-β-actin | Proteintech | 60008-1-Ig |
| Anti-GAPDH | Proteintech | 60004-1-Ig |
| Anti-MLCK | Proteintech | 21642-1-AP |
| Anti-IKKβ | Cell Signaling Technology | #8943 |
| Anti-IKKα | Cell Signaling Technology | #11930 |
| Anti-p-IKKα/β (Ser176/180) | Cell Signaling Technology | #2697 |
| Anti-p-IκBα (Ser32) | Cell Signaling Technology | #2859 |
| Anti-IκBα | Cell Signaling Technology | #4814 |
| Anti-p-p65 | Cell Signaling Technology | #3033 |
| Anti-p65 | Cell Signaling Technology | #8242 |
| VeriBlot for IP Detection Reagent | Abcam | ab131366 |
| Rabbit IgG | Beyotime Biotechnology | A7016 |
| Goat Anti-Rabbit IgG Antibody (H+L), Cy3 Conjugated | Bioss | bs-0295G-Cy3 |
| Anti-mouse IgG,HRP-linked Antibody | Cell Signaling Technology | #7076 |
| Anti-rabbit IgG, HRP-linked Antibody | Cell Signaling Technology | #7074 |
| FITC-dextran | Sigma-Aldrich | 46944 |
| TNF alpha Mouse Uncoated ELISA Kit | Thermo Fisher Scientific | 88-7324-88 |
| IL-6 Mouse Uncoated ELISA Kit | Thermo Fisher Scientific | 88-7064-88 |
| Mouse IFN gamma Uncoated ELISA | Thermo Fisher Scientific | 88-7314-88 |
| Mouse IL-1β ELISA Kit | MULTISCIENCES | EK201B/3 |
| DSS | MP Biomedicals | 9011-18-1 |
| TNBS | Sigma-Aldrich | E0753 |
| IFN-γ | Peprotech | 300-02-20 |
| TNF-α | Peprotech | 300-01A |
| Anti-IKKα | Abcam | ab32041 |
| IKK-16 | Selleck | S2882 |
| BV510 Rat Anti-CD11b | BD Biosciences | 562950 |
| BB700 Hamster Anti-Mouse CD11C | BD Biosciences | 566505 |
| PE Rat Anti-Mouse CD103 | BD Biosciences | 557495 |
| Alexa Fluor® 700 Rat Anti-Mouse CD45 | BD Biosciences | 560510 |
| PE-CF594 Rat Anti-Mouse F4/80 | BD Biosciences | 565613 |
| Fixable Viability Stain 780 | BD Biosciences | 565388 |
| BV605 Rat Anti-Mouse Ly-6C | BD Biosciences | 563011 |
| PE-Cy™7 Rat Anti-Mouse Ly-6G | BD Biosciences | 560601 |
| BB515 Rat Anti-Mouse I-A/I-E | BD Biosciences | 565254 |
| PerCP-Cy™5.5 Hamster Anti-Mouse CD3e | BD Biosciences | 551163 |
| PE Rat Anti-Mouse CD8a | BD Biosciences | 553032 |
| BV510 Rat Anti-Mouse CD19 | BD Biosciences | 562956 |
| CD4 Monoclonal Antibody, FITC | eBioscience | 11-0041-82 |
| CD25 Monoclonal Antibody, APC | eBioscience | 17-0251-82 |
| Anti -CD4 Rabbit pAb | Servicebio | GB11064 |
| Anti -FOXP3 Rabbit pAb | Servicebio | GB11093 |
| Anti -IL-17 Rabbit pAb | Servicebio | GB11110-1 |
| Anti-IFN-γ | Bioss | bs-0480R |
| Anti-IL-4 | Bioss | bs-0581R |

**Supplementary Table 4 The primers and related sequences in the study**

| **Primers and related sequence** | **SEQUCENCE** |
| --- | --- |
| PBLD (human) | Forward: TTGGGAGTTGAGCACCTTCG |
|  | Reverse: TTACGTCTTCTGGGCTGACG |
| TNF-α (human) | Forward: GAACTCACTGGGGCCTACA |
|  | Reverse: GCTCCGTGTCTCAAGGAAGT |
| IL-6 (human) | Forward: CAAAGATGTAGCCGCCCCAC |
|  | Reverse: GCCTCTTTGCTGCTTTCACAC |
| IL-1β (human) | Forward: CAAGCAGAAAACATGCCCGT |
|  | Reverse: AGCACAGGACTCTCTGGGTA |
| IL-8 (human) | Forward: ACACTGCGCCAACACAGAAA |
|  | Reverse:CTGGCATCTTCACTGATTCTTGG |
| ZO1 (human) | Forward: CCAGAAATACCTGACGGTGCT |
|  | Reverse: CAGAGGATGGCGTTACCCAC |
| Occludin (human) | Forward: CACACCTACACTCCCGCGT |
|  | Reverse: GCAATGCCCTTTAGCTTCCAA |
| Claudin1 (human) | Forward: TTGGGCTTCATTCTCGCCTT |
|  | Reverse: GAGGATGCCAACCACCATCA |
| GAPDH (human) | Forward: GACAGTCAGCCGCATCTTCT |
|  | Reverse: GCGCCCAATACGACCAAATC |
| TNF-α (mouse) | Forward: GTAGCCCACGTCGTAGCAAA |
|  | Reverse: ACAAGGTACAACCCATCGGC |
| IL-6 (mouse) | Forward: TTTCCTCTGGTCTTCTGGAGTA |
|  | Reverse: CTCTGAAGGACTCTGGCTTTG |
| IL-1β (mouse) | Forward: GAGGACATGAGCACCTTCTTT |
|  | Reverse: GCCTGTAGTGCAGTTGTCTAA |
| IFN-γ (mouse) | Forward: GGCCATCAGCAACAACATAAG |
|  | Reverse:GTTGACCTCAAACTTGGCAATAC |
| CCL20(mouse) | Forward: AACTGGGTGAAAAGGGCTGT |
|  | Reverse: GTCCAATTCCATCCCAAAAA |
| IL17c(mouse) | Forward: CTCCTGCTTCTAGGCTGGTTG |
|  | Reverse: CCACCTGGCACTTCGAGTTAG |
| GAPDH (mouse) | Forward: GGAGAAACCTGCCAAGTATGA |
|  | Reverse: TCCTCAGTGTAGCCCAAGA |
| ZO1(mouse) | Forward: CATCTCCAGTCCCTTACCTTTC |
|  | Reverse: CCTCCAGGCTGACATTAGTTAC |
| Occludin(mouse) | Forward: GAGCTTACAGGCAGAACTAGAC |
|  | Reverse: CAGCCATGTACTCTTCACTCTC |
| Claudin1(mouse) | Forward: GGTTATCGGAACTGTGGTAGAA |
|  | Reverse: GTGCTCAGGGAAGATGGTAAG |

**Supplementary Table 5 sgRNA sequence targeting Pbld1&Pbld2 genes**

| **sgRNA** | **Sequence (5’-3’)** | **PAM** |
| --- | --- | --- |
| Pbld1&Pbld2-5’ | TGAGTGAGGGCCATGGCCGA | AGG |
| Pbld1&Pbld2-3’ | GTGTCTGGAGCGCTGCGGTC | TGG |

**Supplementary Table 6 Detailed scoring method of Disease Activity Index**

| **Score** | **Weight loss** | **Stool character** | **Occult or rectal bleeding** |
| --- | --- | --- | --- |
| 0 | No change | Normal | Negative |
| 1 | 1-5% |  |  |
| 2 | 5-10% | Soft stool | Positive occult blood in stools |
| 3 | 10-20% |  |  |
| 4 | >20% | Diarrhea | Bloody stools |
